# An Automated Lab‐On‐A‐Chip Approach for Pollen Tube Growth Manipulation in a Controlled Chemical Environment

**DOI:** 10.1002/advs.202507434

**Published:** 2025-09-29

**Authors:** Jiawei Zhu, Marta Belloli, João P. Vale, Vitaly Pustovalov, Peter Fischer, Hannes Vogler, Tiago S. Mayor, Salvador Pané, Semih Sevim, Ueli Grossniklaus, Bradley J. Nelson

**Affiliations:** ^1^ Multi‐Scale Robotics Lab Institute of Robotics and Intelligent Systems ETH Zurich Tannenstrasse 3 Zurich 8092 Switzerland; ^2^ Department of Plant and Microbial Biology & Zurich‐Basel Plant Science Center University of Zurich Zollikerstrasse 107 Zurich 8008 Switzerland; ^3^ Transport Phenomena Research Centre (CEFT) Faculty of Engineering University of Porto Rua Dr. Roberto Frias Porto 4200‐465 Portugal; ^4^ Associate Laboratory in Chemical Engineering (ALICE) Faculty of Engineering University of Porto Rua Dr. Roberto Frias Porto 4200‐465 Portugal; ^5^ Institute of Food Nutrition and Health ETH Zurich Schmelzbergstrasse 9 Zurich 8092 Switzerland

**Keywords:** automated single cell experiment, controlled chemical gradients, lab automation, lab‐on‐a‐chip, microfluidics, pollen tubes, single cell manipulation

## Abstract

Laboratory automation is successfully implemented across a wide range of applications, from space exploration to oceanic research, facilitating data collection and analysis while improving precision in biological and medical fields. The future of robotic laboratory automation is closely tied to advancements in miniaturization. Thus, automation of lab‐on‐a‐chip (LoC) systems–integrating complex laboratory tasks onto a small chip–holds great potential for scientific research, including the study of model organisms and cells. Here, an automated continuous‐flow‐based LoC device designed to investigate and manipulate the growth of pollen tubes (PTs)–fastest‐growing cells in nature–within controlled chemical environments is presented. The automated LoC approach allows for the generation of tailored chemical gradients (e.g., of Ca^2+^) around the PT tip, offering unprecedented precision and efficiency in the manipulation of PT growth when compared to manual experiments. Besides advancing the experimental methodology by providing more precise information on the response of PTs to Ca^2+^ concentration gradients, the developed closed‐loop approach with simultaneous data recording and processing reduces the time and costs associated with experiments. This underscores the great potential of robotic laboratory automation for streamlining data collection and analysis, paving the way for more efficient and precise scientific research.

## Graphical abstract

Here, a microfluidic‐based robotic lab‐on‐a‐chip (LoC) device is presented for automated, continuous‐flow investigation and manipulation of single pollen tube growth under precisely controlled chemical gradients. This closed‐loop system streamlines data collection and analysis while enhancing experimental precision compared to manual methods, highlighting the transformative potential of robotic laboratory automation for miniaturized, cost‐effective single‐cell research.

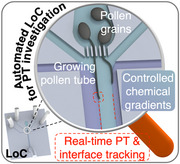

## 1 Introduction

Laboratory automation has emerged as a significant ongoing research topic in recent years.^[^ ^1^, ^2^, ^3^, ^4^ ^]^ Large‐scale automation has found a wide range of uses, for example, facilitating data collection in space,^[^ ^5^ ^]^ enabling automatic observations in the depths of oceans,^[^ ^6^, ^7^ ^]^ and contributing to the recent discovery of dark oxygen.^[^ ^8^ ^]^ Beyond helping in collecting and analyzing data from difficult‐to‐reach locations, automated robotic systems have proven invaluable in medical fields, assisting surgeons and scientists in medical applications with tasks demanding exceptional precision and dexterity.^[^ ^9^, ^10^, ^11^ ^]^ Moreover, laboratory automation, such as the use of robots to collect and analyze data, plays a key role in acquiring the required datasets with enhanced precision, especially once it is combined with the power of artificial intelligence (AI). While the integration of robotics and AI is still evolving, this promising partnership has already made a significant contribution to the advancement of laboratory automation, paving the way for self‐driving laboratories capable of discovering new materials^[^ ^12^, ^13^, ^14^ ^]^ and drugs^[^ ^15^, ^16^ ^]^ with greater efficiency, enhanced precision, and minimal human intervention.

The future of robotic laboratory automation is closely tied to advancements in the miniaturization of experimental setups.^[^ ^17^ ^]^ In this vein, microfluidics is a key technology as it enables the precise manipulation of tiny amounts of fluid through micron‐sized channels.^[^ ^18^ ^]^ For example, integrated microdevices for reaction automation have revolutionized the optimization of organic reactions,^[^ ^19^ ^]^ pharmaceutical production,^[^ ^20^ ^]^ and peptide synthesis^[^ ^21^ ^]^ by incorporating in‐line characterization techniques within closed‐loop systems and using machine learning to design synthesis routes.^[^ ^22^ ^]^ Microfluidic devices can also be employed in biological research as lab‐on‐a‐chip (LoC) or organ‐on‐a‐chip (OoC) systems. These compact and portable setups streamline and automate repetitive laboratory tasks, a feature that can be further enhanced with the integration of machine learning to create miniaturized self‐driving laboratories.^[^ ^23^, ^24^ ^]^ These integrated microfluidic systems have been successfully applied in cell sorting,^[^ ^25^ ^]^ real‐time flow cytometry,^[^ ^26^, ^27^ ^]^ point‐of‐care infectious disease diagnostics,^[^ ^28^ ^]^ and combinatorial drug screening.^[^ ^29^, ^30^ ^]^ Furthermore, high‐throughput and automated LoC devices are also appealing tools for space research, significantly reducing payload and launch costs.^[^ ^31^ ^]^

Microfluidic‐based LoC systems are advanced microengineering tools that provide precise manipulation, immobilization, automated alignment, sorting, sensory, and mechanical and chemical stimulation capabilities. These features significantly advance research in model organisms, such as *Caenorhabditis elegans* (nematode)*, Drosophila melanogaster* (fruit fly)*, Danio rerio* (zebrafish), among others.^[^ ^32^ ^]^ Pollen tubes (PTs) are compelling model systems because of their rapid and polarized tip growth, which distinguishes them as the fastest‐growing single plant cell.^[^ ^33^, ^34^, ^35^, ^36^ ^]^ This characteristic makes PTs ideal specimens for single cell manipulation studies.^[^ ^37^, ^38^, ^39^ ^]^ A PT can precisely navigate to the female gametophyte by directional cue sensing.^[^ ^40^, ^41^ ^]^ Specifically, both intracellular and extracellular Ca^2+^ signaling have been identified as significant contributors to PT growth and directionality.^[^ ^42^, ^43^ ^]^ Research on the chemotropic behavior of growing PTs have been typically conducted in simple culture mediums within containers.^[^ ^43^, ^44^, ^45^ ^]^ This approach not only hinders the selection and observation of individual cells, but also makes it impossible to precisely control the external conditions, such as Ca^2+^ concentration, and, thus, obtain reliable quantitative data on how external conditions impact PT growth. While tip‐growing cells have also been investigated within LoC devices incorporating chemical gradients, microstructural features and integrated biosensors,^[^ ^46^, ^47^, ^48^ ^]^ these studies often lack statistical data and sufficient replication, which are essential for a comprehensive understanding of biological responses. For that reason, the implementation of automated LoC approaches could successfully eliminate the aforementioned problems by facilitating precise data collection and statistical analysis. So far, the closed‐loop automation of microfluidic systems has shown great promise in various tasks, including droplet formation,^[^ ^49^ ^]^ fluidic output regulation,^[^ ^50^ ^]^ and measuring and controlling cell flow speeds with integrated sensors.^[^ ^51^ ^]^ However, it has not yet been applied to manipulating and investigating the growth of PTs, which are tip‐growing single cells.

**Figure 1:**
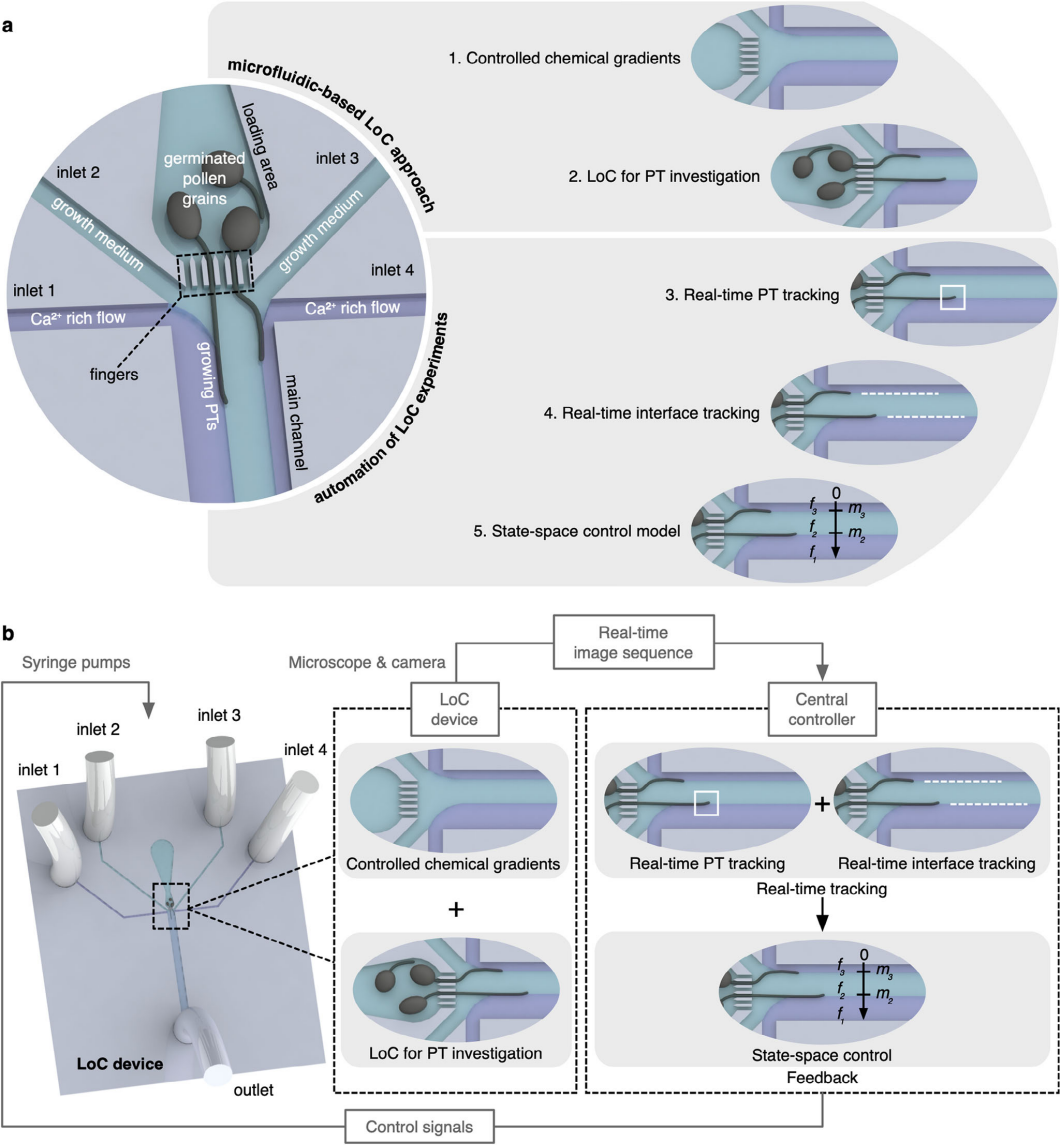
Conceptual illustrations of our automated Lab‐on‐a‐chip (LoC) approach to investigate and manipulate the pollen tube (PT) growth in a controlled chemical environment. a) Schematics showing the highlighted features of a microfluidic‐based LoC approach and automated experiments. b) Workflow showing the automation and closed‐loop control of the integrated system. In the state‐space control model, *f_1_*, *f_2_*, and *f_3_* represent the flow rates of three co‐flowing streams, while *m_2_* and *m_3_* are the positions of the laminar flow interfaces between them (vide infra).

In this study, we present an advanced automated LoC approach for investigating and manipulating the growth of single PTs within precisely controlled chemical environments, including customized Ca^2+^ gradients. A continuous‐flow‐based LoC device facilitates the generation and localization of controlled Ca^2+^ gradients around the PT tip. Automation is achieved through real‐time tracking of the PT tip and the interfaces between different streams, with state‐space‐model‐based closed‐loop control facilitating autonomous operation of self‐driving LoC experiments (**Figure** **1**). This automated system offers exceptional control and high efficiency in the manipulation of PT growth, significantly enhancing the precision of the statistical data collected. Such precision is essential for accurately correlating PT responses to the local lateral concentration differences at the tip (i.e., the actual concentrations sensed by the PT). These correlations are comprehensively investigated with numerical mass‐transport simulations. Additionally, by integrating simultaneous data recording and processing, the automation approach developed for biological LoC experiments significantly reduces labor costs and time, highlighting the potential of robotic laboratory automation for data collection and analysis in microfluidics.

## 2 Results

### 2.1 Design of LoC Devices to Control Chemical Gradients Around a Single Pollen Tube

To control the chemical (e.g., calcium) gradients around the tip of a single PT, we adopted a continuous flow‐based microfluidic approach where we can spatiotemporally control the Ca^+2^ concentrations formed around the tip of a single PT (Figure S1a,b, Supporting Information). We selected a 4‐inlet‐based microfluidic device design to achieve a precise control on the localization of the interface positions between co‐flowing laminar streams, and the diffusion time of chemical species by tailoring the relative and total flow rates, respectively (vide infra). The designed LoC devices contain a pollen grain (PG) loading area with a height larger than that of the main channel to fit a large number of PGs without crushing them which would increase the probability of germination, as well as PT growth fingers to limit the number of PTs growing into the main channel (Figures S1b, S2, Supporting Information, see also Section 4 for more details). This distinctive LoC design with 4 inlets allows for the precise localization of controlled concentration gradients around the tip of growing pollen tubes in the main channel where co‐flowing growth medium streams with different chemical compositions–, i.e., solutions either high or low in Ca^2^⁺, relative to the optimal growth medium (which contains 130 µm Ca^2^⁺)–form laminar flow interfaces.

To characterize the hydrodynamic performance of the designed LoC devices, we performed hydrodynamic flow focusing experiments using Ca^2^⁺‐rich flows (from inlets 1 and 4) and Ca^2^⁺‐poor flows (from inlets 2 and 3), resulting in two distinct interfaces (i.e., interface 1 and 2) whose positions can be accurately controlled by tailoring the flow rate ratio (FRR) and the asymmetric flow rate ratio (AFRR) (Figure S1c–f, Supporting Information). FRR is defined as the ratio of the middle stream flow rate (i.e., the sum of the flows from inlet 2 and 3, *v*_2_ + *v*_3_) to the total flow rate (TFR, i.e., the sum of flows from all four inlets, $\sum_{i=1}^{4}v_{i}$; *FRR*  = (*v*_2_ + *v*_3_)/*TFR* ). On the other hand, the AFRR is the ratio of the sum of the flow rate of inlet 4 and half of the flow rate of the middle stream, to the TFR (i.e.*, AFRR*  = (*v*_4_ + 0.5(*v*_2_ + *v*_3_))/*TFR* ). Specifically, the FRR specifies the focusing level of the middle stream and the AFRR indicates the level of asymmetry in the flow rates of the two lateral streams. This configuration enables to simultaneously control the width and center line position of the middle stream via changes in FRR and AFRR, respectively, and consequently, the precise location of each interface (Figure S1g, Supporting Information). By leveraging the relationship between the interface position and relative ratio of the flow rates, we were able to accurately position each interface as desired (spatial control, Supporting Movie S1, Supporting Information). This linear relationship, which primarily holds across the width of the main channel–except when interfaces are too close to the side walls where “*no‐slip”* conditions prevail–was effectively used for positioning the interface during the automated LoC experiments (vide infra). Moreover, independently adjusting the TFR allows for precise control over both the diffusion time of chemical species (temporal control) and the hydrodynamic conditions (such as pressure and shear rates) within the main channel, without affecting the position of the interfaces (Figure S1h, Supporting Information). This is a key feature that allows for precise regulation of the pressure exerted on the PTs during LoC experiments, thus avoiding harmful high pressures. By maintaining a constant TFR and adjusting the relative flow rates, the interfaces can be repositioned along the width of the main channel to align with the tip of the growing PT, without altering the pressure around the PT tip. This approach helps to prevent undesired sliding of the PT caused by transient pressure fluctuations.

### 2.2 Single Pollen Tube Manipulation in LoC Devices

**Figure 2:**
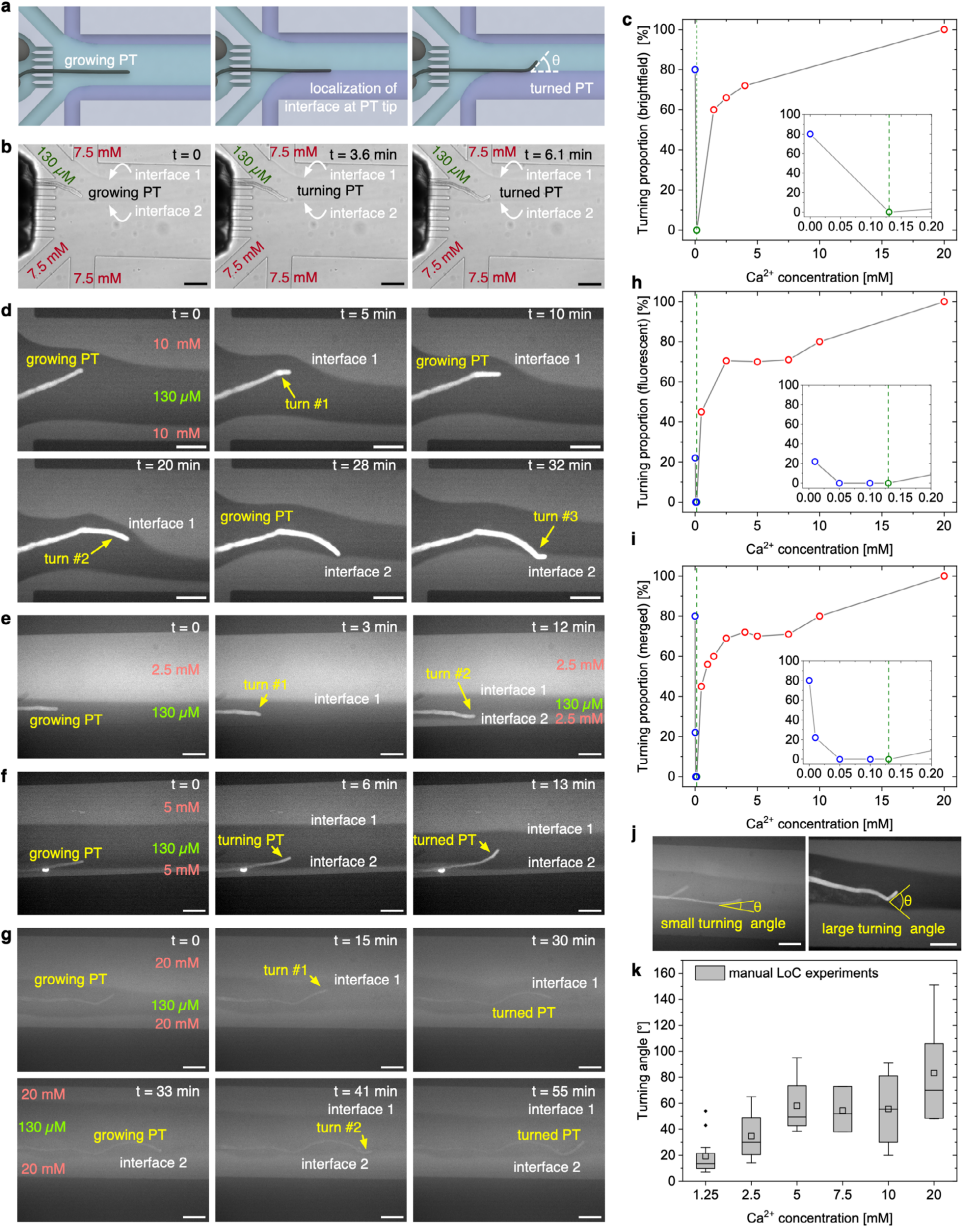
Manual LoC experiments to investigate and manipulate the PT growth under controlled Ca^2+^ gradients. a) Schematic illustration of LoC experiments. b,d–g) Image sequence showing the representative turning behavior of growing PT tip when exposed to the interfaces between laminar streams having different Ca^2+^ concentrations, investigated under b) brightfield and d–g) fluorescence microscopy. PT was subjected to the interface between the optimum (130 µm) and calcium rich growth medium, b) 7.5 mm, d) 10 mm, e) 2.5 mm, f) 5 mm, and g) 20 mm of Ca^2+^ concentration, respectively. (see also Supporting Movies S2–S6, Supporting Information) c,h,i) Statistical analysis showing the correlation between PT turning proportion and Ca^2+^ concentration of the side streams, obtained from experiments performed under c) brightfield and h) fluorescence microscopy, and i) merged results including all experiments in both brightfield and fluorescence (n ≥5 for each datapoint in c) and h), and n ≥10 for each data point in i)). Insets show the magnified region between 0 and 0.2 mm Ca^2+^. Results of the experiments performed with growth mediums having poor, optimum and rich calcium content are color coded with blue, green, and red circles, respectively. j) Examples showing the different responses of PTs to the interface, e.g., a small and large turning angle. k) Box plot showing the correlation between PT turning angle (for all first turns avoiding high calcium) and Ca^2+^ concentration of the side streams. The graph shows the results of 34 individual experiments. The box, whiskers, square, line and dots represent the range of 25%‐75% of the data, 1.5 inter quartile range, mean, median and outliers of the data (n ≥3), respectively. Scale bars are 100 µm.

Continuous flow‐based LoC devices were initially employed to investigate and manipulate the growth of *Lilium longiflorum* (lily) PTs under controlled Ca^2+^ gradients, because of their relatively high germination rate and rapid growth in vitro.^[^ ^39^ ^]^ To benchmark our study and evaluate the performance of our LoC devices for in vitro PT manipulation experiments within controlled chemical environments, we initially conducted brightfield experiments with lily PTs, manually controlling the flow rates via a syringe pump to position the interfaces at the PT tip. In a typical experiment, the lily PGs were loaded into the LoC device containing a growth medium with optimal Ca^2+^ concentration (i.e., 130 µm ^[^ ^52^ ^]^). Following the germination of a single PT in the main channel, growth mediums with varying concentrations of Ca^2+^ were pumped from separate inlets to form laminar flow interfaces and establish Ca^2+^ gradients. The direct interaction of PTs with these flowing streams allows for precise positioning of sharp concentration gradients around the PT tip. As illustrated in **Figure** **2**a, by manually adjusting the relative flow rates of different streams (while maintaining a constant TFR between 6 and 10 µL min^−1^), the interface between the flow with the optimal Ca^2+^ concentration and flows with either higher or lower Ca^2+^ concentrations could be positioned at the tip of PT. This manipulation induced changes in the growth direction of the PT (i.e., turning), effectively steering it away from non‐ideal concentrations. Note that because of the change in the refractive index, interfaces between the growth media with different calcium concentrations are visible under brightfield microscopy,^[^ ^53^ ^]^ which allows for accurate positioning and adjustment of the interface by regulating the flow rates (Figure 2b; Supporting Movie S2, Supporting Information). Statistical analysis revealed that a higher proportion of the growing PTs displayed a turn with increasing Ca^2+^ concentration of the side streams. At 20 mm, 100% of the PTs turn away to avoid the unfavorably high Ca^2+^ concentration (Figure 2c, red circles). When Ca^2+^‐free growth medium was flowed in the side streams with the optimal Ca^2+^ concentration (130 µm) in the middle stream, ≈80% of the PTs turned, indicating their tendency to avoid Ca^2+^‐poor environments (Figure 2c, blue circle). In contrast, control experiments with optimal growth medium in all streams resulted in no turning events, confirming that the PT tip was responding to calcium concentration, rather than changes in the relative flow rates between the different inlets (Figure 2c, green circle).

We then focused on LoC experiments performed with fluorescence microscopy which, compared to the brightfield microscopy, offers an enhanced visualization of the interfaces between co‐flowing streams even if having only slight differences in Ca^2+^ concentrations, as well as more precise quantification of Ca^2+^ levels within the LoC devices. Additionally, data from fluorescent biosensors could also provide physiological and biochemical information about growing PTs in situ.^[^ ^54^, ^55^ ^]^ To this end, we adapted our approach to fluorescence microscopy. To observe the PTs and co‐flowing streams using fluorescence microscopy, we respectively stained them with the cell‐permeant calcium indicator CaGreen,^[^ ^56^ ^]^ which operates intracellularly, and the fluorescent dye Fluo‐3, which detects free Ca^2+^ in the growth medium.^[^ ^57^ ^]^ This staining strategy enables effective fluorescence‐based investigations of growing PT within the LoC device, and allows for manipulation of the growth (i.e., inducing multiple turning events in a single PT) by exposing different sides of the growing tip to unfavorable Ca^2+^ concentrations (Figure 2d–g; Supporting Movies S3–S6, Supporting Information). As shown in Figure 2h, statistical analysis of fluorescent experiments yields results consistent with those from experiments conducted with brightfield microscopy. This indicates that the use of CaGreen and Fluo‐3 in LoC experiments does not affect PT germination, growth or turning. In response to calcium concentration, the proportion of PTs showing turning displayed similar patterns with and without the use of these dyes (comparing Figure 2c–h). Therefore, combining data from both methodologies enables a robust and statistically comprehensive understanding of PT growth under controlled gradients of Ca^2+^ concentration.

The analysis of LoC experiments (Figure 2i) across varying Ca^2+^ concentrations (from 0 to 20 mm) in the side streams reveals that the proportion of PTs showing turning increases with increasing Ca^2+^ concentration, reaching 45%, 60%, 70%, and 80% for Ca^2+^ concentrations of 0.5, 1.5, 5, and 10 mm, respectively. Moreover, 100% of the PTs turned when they were exposed to the interface between the growth mediums having optimal (0.13 mm) and 20 mm Ca^2+^ concentration (Figure 2i, red circles). Using Ca^2+^ concentration of 0.01 mm in the side streams results in turn events in 22% of the cases, whereas using growth medium without calcium results in turn events in 80% of the cases (Figure 2i, blue circles). This suggests that PTs exposed to interfaces between calcium‐poor solutions and the growth medium with an optimal calcium concentration also exhibit high number of turning events to avoid calcium‐depleted regions. Conversely, PTs do not display turning events in growth media with Ca^2+^ concentrations between 0.05 and 0.13 mm, suggesting that these concentrations are well‐tolerated by the PTs (Figure 2i, blue and green circles). Overall, PTs exhibit avoidance responses to both media with insufficient and excessive Ca^2+^ by displaying more turning events with the higher concentration difference exposed to both sides of the PT tip.

In addition to examining the proportion of PTs displaying turning, we also investigated the turning angle of the PTs (Figure 2j), which varies depending on the concentration gradients around the PT tip. Our findings indicate that the turning angle increases with increasing concentration difference. Specifically, the average turning angle increases from ca. 20° to over 80° as the Ca^2+^ concentration in the side streams increases from 1.25 to 20 mm. However, at higher Ca^2+^ concentrations (≥10 mm), we observed a large variation in turning angle between individual experiments (Figure 2k; for more details, see also Section 2.3).

### 2.3 Numerical Mass‐Transport Simulations

To understand the above‐mentioned phenomena, we performed numerical simulations of the fluid flow and mass transport within the LoC devices, to assess the local concentration gradients formed around the PT tip under continuous flow conditions. First, we evaluated the parameters required for the simulations (i.e., density and rheology of growth mediums, as well as the diffusion coefficient of calcium in growth mediums) and validated our simulation approach for capturing the physics at play (for more details see also Section 4 and Figures S3–S5, Supporting Information).

**Figure 3:**
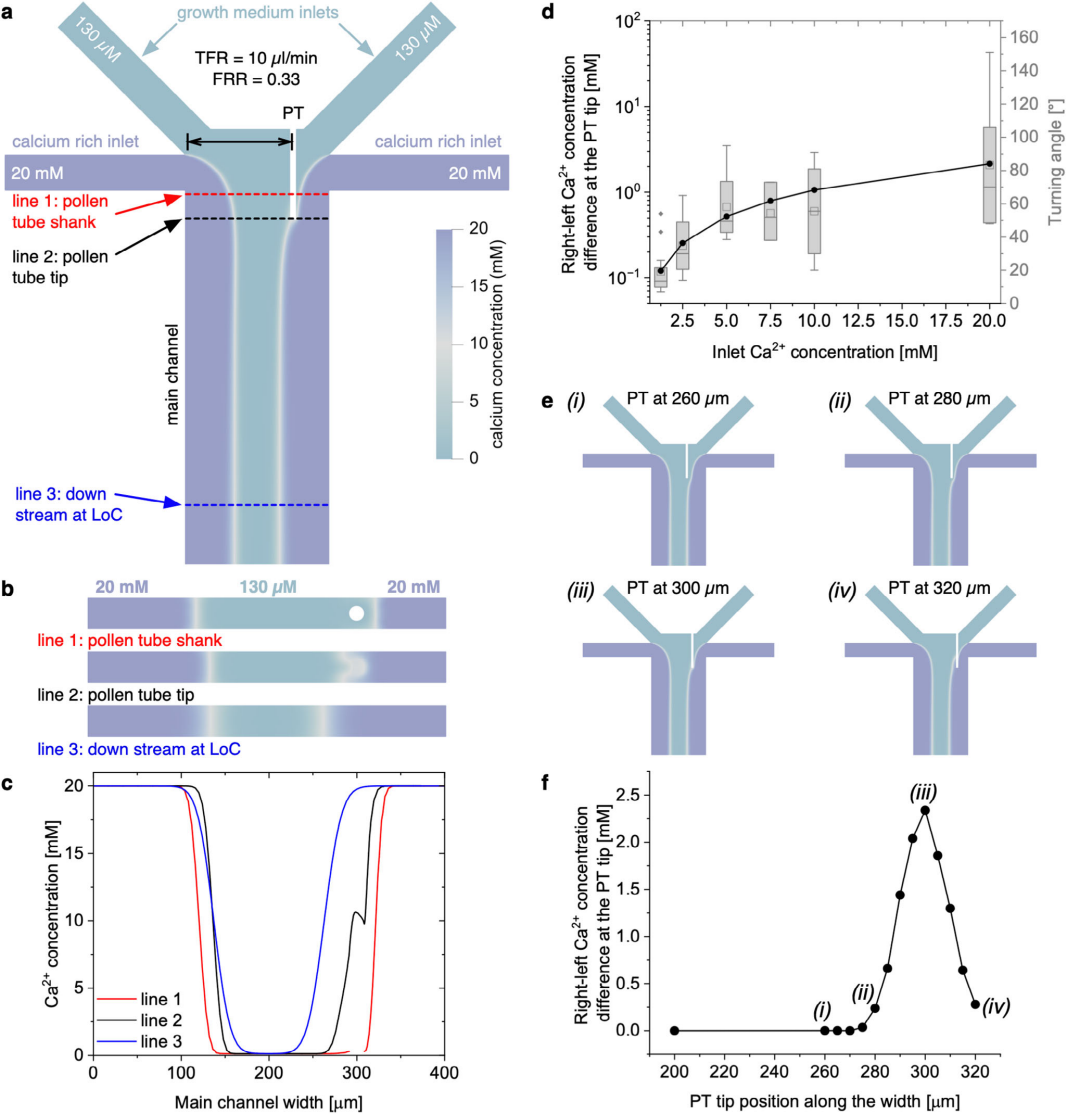
Numerical simulations to investigate the Ca^2+^ gradients around the PT tip. a) Concentration maps showing the Ca^2+^ distribution in the LoC device when the optimal (130 µm) and calcium rich (20 mm) growth mediums were injected through middle (inlets 2 and 3) and side (inlets 1 and 4) inlets, respectively, attaining a FRR of 0.33 (AFRR of 0.5) at TFR of 10 µL min^−1^. b) Concentration maps showing the Ca^2+^ distribution at different cross‐sections, marked in panel a). c) Graph showing the Ca^2+^ concentration profiles at the middle height of the main channel considering three different locations along the length of the channel. d) Simulated right‐left Ca^2+^ concentration difference at the PT tip (black line and dots) to compare with the experimentally obtained turning angles at different inlet Ca^2+^ concentrations. e) Simulations performed in conditions similar to those in panel a) while changing the position of PT with respect to the interface. f) Resulting right‐left Ca^2+^ concentration differences based on different PT positions along the width of the channel.

After validating the numerical approach, we performed 3D fluid flow and mass transport simulations to investigate the local Ca^2+^ concentration differences in both sides of the PT tip when the interface between the two growth media, i.e., those containing optimum (130 µm) and rich (20 mm) Ca^2+^ concentrations, is located at the tip of the PT (**Figure** **3**a). Specifically, we considered a scenario mimicking the experimental conditions when the optimal and calcium rich growth media were injected through the middle (inlets 2 and 3) and the side (inlets 1 and 4) inlets, respectively, to generate a FRR of 0.33 at TFR of 10 µL min^−1^. Moreover, the PT was modelled as a 250 µm‐long cylindrical tube having a diameter of 17 µm (average diameter of lily PTs^[^ ^39^ ^]^) with its center positioned at the middle height of the device (17.5 µm from the top/bottom wall), such that the tip of the PT is located at the interface between the growth mediums having 130 µm and 20 mm Ca^2+^.

To better understand the mass transport within the LoC device, we investigated local Ca^2+^ concentrations at different cross‐sections along the microfluidic channel, particularly focusing on three key regions: the PT shank (line 1), the PT tip (line 2), and the downstream of the LoC device (line 3) (Figure 3a). While the interface near to the PT's shank part (line 1) was slightly displaced sideways because the presence of the pollen tube affected the hydrodynamics, both interfaces at the downstream area (line 3) were symmetrically positioned as expected from the hydrodynamic flow focusing conditions (Figure 3b,c). More importantly, numerical results around the tip part (line 2) showed that the formation of local Ca^2+^ gradients–modified by the presence of the PT–results in a concentration difference between both sides (right and left) of the PT tip (Figure 3b,c; Figure S6a, Supporting Information), where the PT is actually growing from and is most sensitive to its environment.^[^ ^58^ ^]^ Note that these local Ca^2+^ gradients around the PT tip can be generated regardless of tip position within the 3D domain of the main channel (e.g., vertical: close to the top/bottom walls; lateral: close to the side walls or middle of the main channel; and longitudinal: close to the downstream of the main channel) by simply adjusting the relative flow rates (Figures S7,S8, Supporting Information). The numerical simulations demonstrate that the Ca^2+^ concentration profiles and, thus, the PT growth and occurrence of turning events can be precisely controlled by adjusting the flow rates in the LoC device, irrespective of the PT's position or length (Figure S7,S8, Supporting Information).

To mimic other experimental conditions tried in the LoC device, we performed further simulations with different Ca^2+^ concentrations (ranging from 1.25 to 20 mm) in the calcium‐rich inlets. The difference in Ca^2+^ concentration between the right and left sides of the PT tip increases with rising Ca^2+^ concentration in the calcium‐rich inlets, in line with what was observed for the turning angle (Figure 3d). These results suggest that the concentration difference between both sides of the tip sensed by the pollen tube plays an important role in the turning events, modulating its response in terms of the growth direction and turning angle. Yet, this is insufficient to explain the large variation in turning angle observed between individual experiments, especially for high Ca^2+^ concentrations. With the aim of clarifying this issue, we investigated how local concentration differences experienced by a PT vary with the distance between its tip and the interface. In manually controlled LoC experiments, turning events seemed to occur at different distances from the interface (Figure S9 and Supporting Movie S7, Supporting Information), likely due to variability introduced by the human operators manually adjusting the relative flow rates to control the interface position. The local concentration differences can drastically change depending on the position of the interface relative to the tip of PT (Figure 3e; Figure S6b, Supporting Information). As seen in Figure 3f, the Ca^2+^ concentration difference between the right and left sides of the PT tip is highest (ca. 2.3 mm) when the PT tip is at the exact position of the interface between the growth media having 130 µm and 20 mm Ca^2+^. This difference quickly decreases to 0 mm as the tip moves away from the interface, in a bell‐shaped curve depending on the distance to the interface (within ±30 µm). These results suggest that any imprecision in manual control of the interface‐to‐tip distance leads to significant variations in the concentration difference sensed by PTs, thereby introducing large deviations in their turning response.

### 2.4 Automation of LoC Devices for Investigating PT Growth

Recognizing the importance of precisely controlling the interface position during the growth of PTs in the LoC device, we developed an automated setup to improve the accuracy of the LoC experiments. We designed the setup to eliminate the variability introduced by the manual control (i.e., of the flow rates) and to reduce the operational time and labor costs associated to data collection and analysis. The automated LoC system was designed to continuously monitor both the PT growth and the position of the interface, dynamically adjusting the flow rates from the syringe pump to maintain a constant distance between the interface and the PT tip. This was effectively achieved through a closed‐loop system that integrates multiple components including the syringe pump, the LoC device, the microscope camera, and the computer.

**Figure 4:**
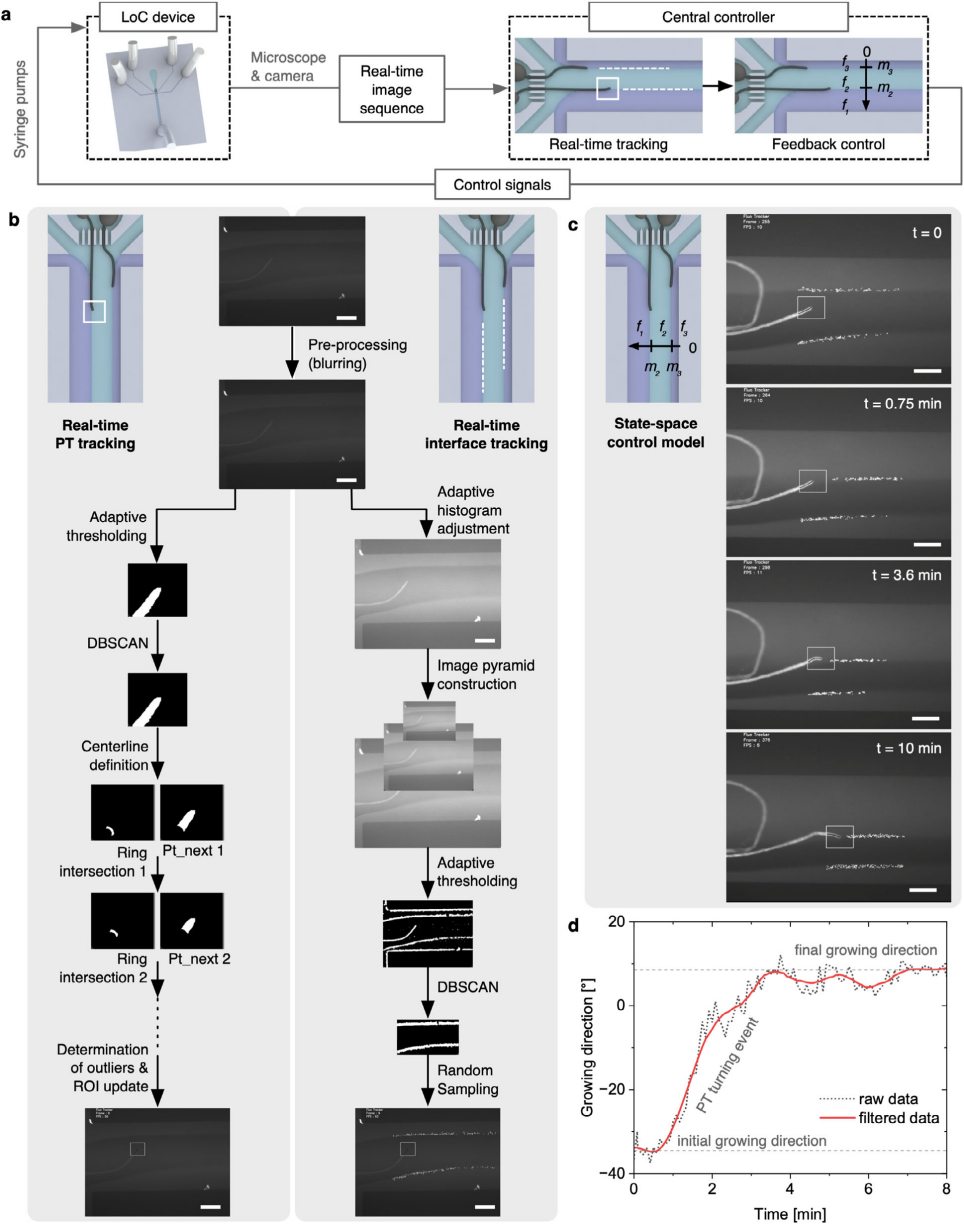
Automated LoC approach to investigate and manipulate the PT growth under controlled Ca^2+^ gradients. a) Illustration showing the workflow of the automated setup. b) Real‐time PT and interface tracking pipelines under fluorescence microscopy. c) Representative image sequence demonstrating the automated LoC experiments utilizing growth mediums having 130 µm and 10 mm Ca^2+^ in middle and side streams, respectively. Scale bars in panels b,c are 100 µm. See also Supporting Movie S9 (Supporting Information). d) Fluorescence PT tracking results corresponding to the automated LoC experiment in c), demonstrating collected and processed growing direction data by the tracker in real‐time experiments.

As shown in **Figure** **4**a, the system integration for the automation of LoC experiments was established in the LabVIEW software. The integrated system consists of several interconnected modules that form a fully automated setup, controlled via a user‐friendly graphical user interface (GUI). At the core of the system, a LabVIEW program coordinates the communication between the microscope camera, the syringe pumps, and a central controller to manage tracking and automation. The microscope equipped with a camera continuously monitors the PT growth within the LoC device. The real‐time image feed is sent to the LabVIEW program, which interfaces with a Python‐based central controller (PCC), for tracking and analysis. The PCC functions as a tracking module, analyzing image sequences to extract key information, such as PT tip position, growth orientation, and the shape and position of the interfaces. Acting as the cognitive center, the PCC calculates the feedback control inputs based on these observations. LabVIEW then receives these inputs and sends control signals to the syringe pumps to adjust the flow rates, thereby altering the interface positions within the LoC device. This adjustment is captured by the microscope, initiating the next cycle and completing the closed‐loop control system for precise tracking and manipulation of PT growth under controlled concentration gradients.

Although we initially developed a brightfield tracking module to analyze PT responses in manually controlled LoC experiments (see Figures S10,S11, Supporting Movie S8, Supporting Information, and Section 4 for details), automation was implemented using fluorescence microscopy as it provides more detailed information on the PT growth and offers clearer real‐time tracking. The fluorescence‐based tracking system uses advanced techniques, such as adaptive thresholding and clustering algorithm “Density‐Based Spatial Clustering of Applications with Noise” (DBSCAN),^[^ ^59^ ^]^ leveraging machine learning to accurately localize the PT tip. Specifically, the frame from the real‐time sequence is first pre‐processed with Gaussian and subsequently median filters for noise elimination. Next, adaptive thresholding is applied within the region of interest (ROI), together with DBSCAN to precisely detect the PT‐tip‐region based on intensity differences in fluorescence images, while DBSCAN efficiently and robustly filters out non‐PT‐region in case of unclear detection. This allows for further pinpointing the centerline of the pollen tube by a series of ring intersections from the edge of ROI with the PT‐tip‐region, hence analyzing and tracking its growth direction. At the end, the system compares the new detection with previously detected data to exclude outliers and in successful cases centers the ROI at the updated PT tip (Figure 4b, left side). In parallel, real‐time interface tracking identifies the boundaries between streams of varying Ca^2+^ concentrations for feedback control. This process involves the adaptive adjustment of the image histogram based on its shape to enhance the contrast, as well as the construction of an image pyramid to gather information at multiple scales, capturing both coarse structures and fine details. Images at various levels are processed with adaptive thresholding and then combined for better representation of the interfaces. The clustering algorithm filters out noises and selects the correct interface curves represented by random sampled points to both leverage precise representation and facilitate data storage, while DBSCAN's robust clustering performance ensures accurate interface detection, which is essential for effective feedback control (Figure 4b, right side).

Besides tracking, the PCC is also responsible for calculating the control inputs based on observations. To this end, a classical state‐space model was developed by non‐dimensionalization of the main channel width along a one‐dimensional axis of range [0,1], and by introducing the state variable (Equation 1) that represents the position of two interfaces (*m*_2_, *and* *m*_3_) formed within the LoC device between three streams. The flow rates of these streams (*f*_1_,*f*_2_,*and* *f*_3_) can be directly controlled by the syringe pumps (Figure 4c). Since our aim was to maintain the pressures and shear rates exerted on the PT constant during the LoC experiments by keeping the TFR unchanged, constraints (Equations (2), (3)) were imposed on the flow rates to ensure these conditions remain stable throughout the experiments.

(1) $$x\left(k\right)=\left[\begin{array}{c} m_{3}\left(k\right)\\ m_{2}\left(k\right) \end{array}\right]$$

(2) $$f_{1}\left(k\right),f_{2}\left(k\right),f_{3}\left(k\right)\varepsilon \left[0,\;TFR\right],\forall k\in N$$

(3) $$\begin{array}{cc} f_{1}\left(k+1\right)+f_{2}\left(k+1\right)+f_{3}\;\left(k+1\right) & =f_{1}\;\left(k\right)+f_{2}\left(k\right)+f_{3}\;\left(k\right)\\ & =TFR,\forall k\in N \end{array}$$

We defined the input variables (Equation 4) as the changes in *f*_3_ and *f*_1_ such that the system could stabilize at the origin. Accordingly, the change in *f*_2_ can be automatically determined by Equation (3).

(4) $$u\left(k\right)=\left[\begin{array}{c} f_{23}\left(k\right)\\ f_{12}\left(k\right) \end{array}\right]=\left[\begin{array}{c} f_{3}\left(k+1\right)-f_{3}\left(k\right)\\ -\left(f_{1}\left(k+1\right)-f_{1}\left(k\right)\right) \end{array}\right]$$

To set up the mathematical model correlating the flow rates with the interface positions, the positions of the interfaces were assumed to have a linear relationship with the corresponding flow rates (Equations (5), (6), where *b*_2_ and *b*_3_ are constants), verified in characterization experiments (see Figure S1, Supporting Information).

(5) $$m_{3}\;\left(k\right)=b_{3}f_{3}\left(k\right),\forall k\in N$$

(6) $$m_{2}\;\left(k\right)=b_{2}\;f_{2}\left(k\right)+b_{3}f_{3}\;\left(k\right)=m_{3}\left(k\right)+b_{2}f_{2}\;\left(k\right),\forall k\in N$$

Accordingly, we proposed a linear model (Equations (7), (8)), where, ω(*k*) is the process error (caused by inaccuracy of the model), *y*(*k*) is the observation (i.e., observed positions of the interfaces by the tracking module), υ(k) is the observation error (caused by the tracking errors), and the matrix B composes only constants (determined by TFR).

(7) $$x\;\left(k+1\right)=x\left(k\right)+B\cdot u\left(k\right)+\omega \left(k\right),B=\left[\begin{array}{cc} b_{3} & 0\\ b_{3}-b_{2} & b_{2} \end{array}\right]$$

(8) $$y\;\left(k\right)=x\left(k\right)+\upsilon \left(k\right)$$

Moreover, we also applied a Kalman filter for better estimating the states.^[^ ^60^ ^]^ The target point *r*(*k*) contains two variables representing the desired positions of the two interfaces (Equation 9)–one being exactly the tracked location of the PT tip, and the other being a dummy variable–while the error is the difference between the target point and our observation (Equation 10).

(9) $$r\;\left(k\right)=\left[\begin{array}{c} r_{3}\left(k\right)\\ r_{2}\left(k\right) \end{array}\right]$$

(10) $$e\;\left(k\right)=r\left(k\right)-y\left(k\right)$$

In summary, the PCC automatically detects the interfaces and determines which interface to adjust based on the detected position and growth direction of the target PT tip. It then performs proportional‐integral (PI) calculations using the positional error to generate control inputs, ensuring that the flow rate constraints are met. (Equations (2), (3)). To define the hyperparameters for the controller and assess its performance, we initially conducted computational test simulations. These simulations demonstrated that the controller accurately estimates the system's real states, even with added random noise, while stabilizing the system at target points with a fast response, minimal overshoot, and very small deviations (Figure S12a, Supporting Information). Additionally, the control inputs remained around the origin, once the system was stabilized (Figure S12b, Supporting Information) and the sensitivity analysis demonstrated a linear relationship between the relative output change and relative input change, with a ratio of approximately 0.16 (Figure S12c, Supporting Information), indicating that the controller exhibits robust performance.

**Figure 5:**
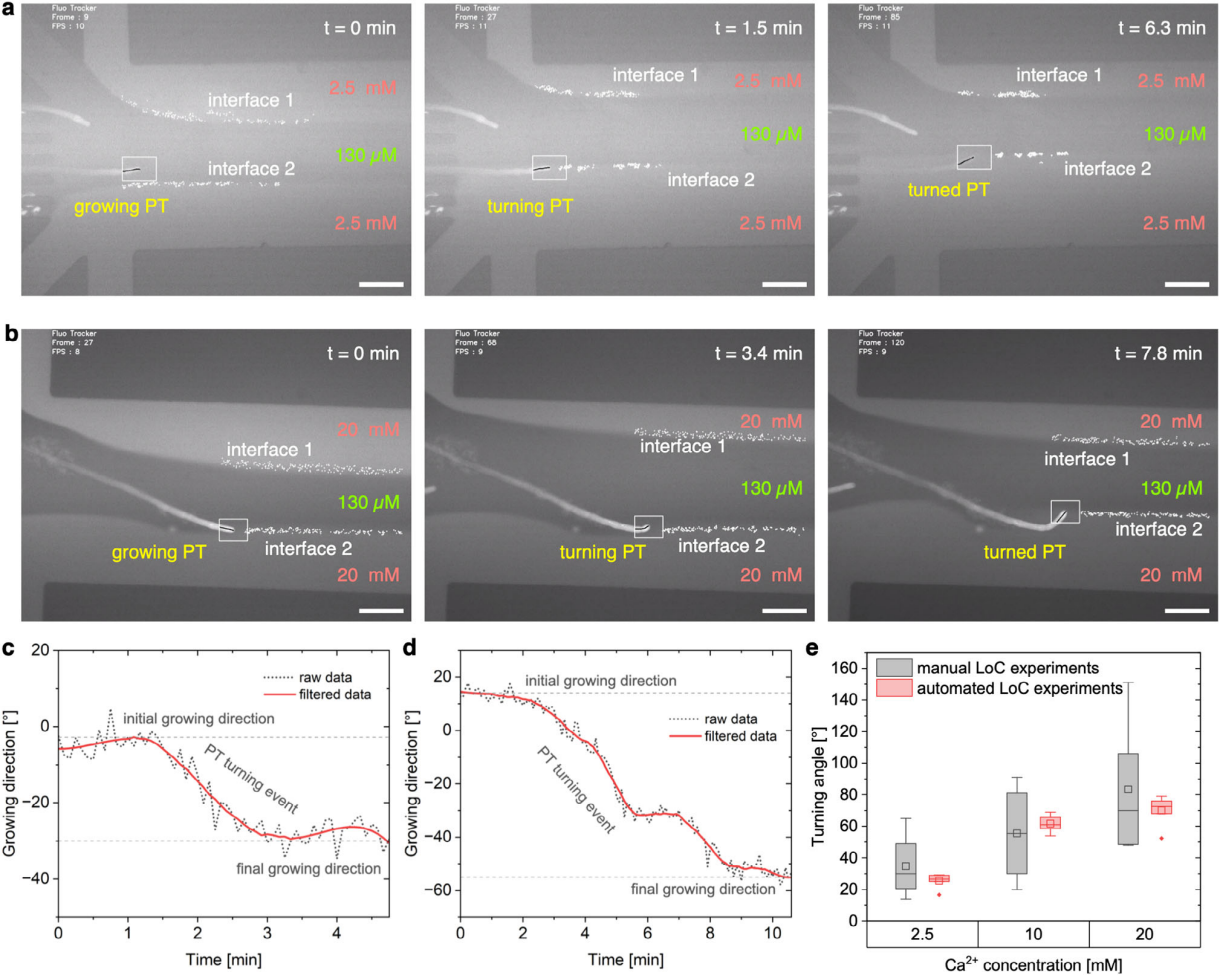
Representative automated LoC experiments, analysis and statistical results for investigating the PT growth under controlled Ca^2+^ gradients. a,b) Image sequence showing the representative turning events of growing PT tip when subjected to the interface between optimum (130 µm) and calcium‐rich growth medium, a) 2.5 mm, and b) 20 mm of Ca^2+^ concentration, respectively. Scale bars are 100 µm. See also Supporting Movies S10–S11 (Supporting Information). c,d) Analyzed and quantified PT growth and turning responses presented in a) and b), respectively. e) Statistical results comparing the manual (grey) and automated (red) LoC experiments. Box plot showing the correlation between PT turning angle (for all first turns avoiding high calcium) and Ca^2+^ concentration of the side streams. The graph shows the results of 15 and 17 individual manual (grey) and automated (red) experiments, respectively. The box, whiskers, square, line and dots represent the range of 25–75% of the data, 1.5 inter quartile range, mean, median, and outliers of the data (n ≥4), respectively.

We then focused on automated LoC experiments to achieve more precise control and manipulation of the PT growth under well‐defined Ca^2+^ gradients. As shown in real‐time automated experiments (Figure 4c; Supporting Movie S9, Supporting Information), after the system was activated at *t* = 0, the upper interface was guided to the PT tip by *t* = 0.75 min and accurately maintained at the tip as the PT turned and grew (*t* = 3.6 min and 10 min, respectively). Throughout the experiments, the PCC continuously recorded and processed critical data, including flow rates, interface shape and position, and the PT tip location and growth direction, enabling streamlined data collection and analysis (Figure 4c,d and **Figure** **5**a–d). To further assess the performance, we conducted automated LoC experiments to manipulate the growth of a single PT under controlled Ca^2+^ gradients, formed at the interface between growth media containing optimal calcium (130 µM) and higher concentrations (2.5 to 20 mm; Figures 4c,d and 5a–e; Supporting Movies S9–S11, Supporting Information). Importantly, the automation not only facilitated data collection and analysis (Figure 5c,d; Figure S13, Supporting Information) but also significantly improved the control over the distance between the PT tip and the interface and, thus, the concentrations sensed by the PT tip. This much higher precision resulted in much smaller deviations in the turning angles, compared to the manually operated experiments (Figure 5e). Specifically, the standard deviations in the turning angles were reduced by 91.3%, 81.4%, and 88.7% at 2.5, 10, and 20 mm Ca^2+^, respectively, leading to an average reduction of 87.1%. These results highlight the effectiveness of the automated LoC setup for achieving more precise and accurate experimental outcomes.

## 3 Conclusion

In summary, our study demonstrates the feasibility and efficacy of LoC devices for investigating the role of Ca^2+^ gradients in regulating the growth of PTs while also highlighting the importance and effectiveness of an automated laboratory setup to improve experimental precision. Through meticulous design and experimentation, we have characterized the sensitivity of PTs to Ca^2+^ concentration gradients, and studied their turning responses to suboptimal Ca^2+^ concentrations. Our automated system approach enabled a significant advancement in the experimental methodology, offering enhanced precision and efficiency in the manipulation of the PT growth within LoC devices. Despite PTs being among the fastest‐growing plant cells in nature,^[^ ^33^, ^34^, ^35^, ^36^ ^]^ studying their growth with LoC experiments traditionally requires several hours and the continuous presence of an operator, making statistical data collection time‐consuming and impractical. In stark contrast, the automation of these biological LoC experiments not only significantly reduces the associated time and labor costs, but also enables simultaneous recording and processing of crucial data with higher precision. This underscores the potential of robotic automation for revolutionizing data collection and analysis in laboratory settings. The insights gained from this work enhanced our understanding of pollen tube growth, laying the groundwork for future research in plant cell signaling and reproductive physiology. Moreover, understanding the mechanisms of plant cell growth and morphogenesis has broader agricultural and economic implications, as plants provide essential raw materials such as food, fiber, wood, and fuel.^[^ ^61^ ^]^ Furthermore, these biological organisms, whose shapes and responses are often mimicked in small‐scale robotics^[^ ^62^ ^]^, offer valuable inspiration for the development of growing small‐scale robots, paving the way for future innovations in robotics.

## 4 Experimental Section

### Materials

Germination medium for *Lilium longiflorum* was prepared according to previous literature^[^ ^52^ ^]^ utilizing 160 µm H_3_BO_3_, 130 µm Ca(NO_3_)_2_, 1 mm KNO_3_, 5 mM MES and 10% (w/v) of sucrose. The pH was adjusted to 5.5 with 0.1N NaOH. To prepare the other types of germination mediums (e.g., calcium poor or rich) used in the LoC experiments, the amount of Ca(NO_3_)_2_ was changed between 0 and 20 mm, while keeping the concentration of the other reagents constant. Note that, to define a suitable concentration range, preliminary tests were conducted before starting the main experiments. At 40 mm Ca^2+^, PTs either burst or failed to grow, making experimentation unfeasible. In contrast, 20 mm was found to be both safe and effective. Therefore, this value was selected as the maximum concentration used in this study. Specifically, the stock solution of germination medium that was prepared without Ca(NO_3_)_2_ was used to dilute the germination medium solution having the highest (20 mm) Ca(NO_3_)_2_ concentration, to precisely prepare germination mediums with other Ca^2+^ concentrations.

### LoC Device Fabrication

First, the dark‐field photolithography masks were designed with AutoCAD software and printed onto polymer film by Selba S.A. Then, master molds were fabricated on silicon wafers via two‐step photolithography and wet etching, using commercial negative photoresists, SU‐8 3025 and SU‐8 100 (micro resist technology GmbH), to accomplish a height of 35 µm in the microfluidic channel area and 130 µm in the pollen grain loading area, respectively (Figure S2, Supporting Information). After fabricating the master molds, polydimethylsiloxane (PDMS) slabs were prepared through replica molding techniques. In this process, PDMS and curing agent (Sylgrad 184, Dow Corning Corporation) were mixed with ratio 10:1, and the mixture was degassed prior to pouring on master molds. After curing the PDMS in oven at 80 °C for 14 h, PDMS slabs were cut and punched for inlet and outlet holes using a 1.5 mm biopsy puncher. Finally, PDMS chips were bonded to a glass slide via oxygen plasma activation and put into an 80 °C oven for 90 min to improve plasma bonding.

### Pollen Germination

*Lilium longiflorum* anthers were collected in 1.5 mL Eppendorf tubes in liquid nitrogen and stored at −80 °C. Prior to LoC experiments, pollen grains were re‐hydrated for 10 min in a humidity chamber and then resuspended in germination medium. They were collected with a syringe and gently inserted in the LoC device through the inlet of pollen grain loading area. Note that, prior to the pollen grain loading, the LoC devices were filled with liquid germination medium (having optimal Ca^2+^ concentration, 130 µm) to avoid air bubbles and overaccumulation of grains in the punched hole inlet. The loaded LoC devices were then kept in a humidity chamber for at least 2 hours, allowing the pollen tubes to germinate and grow in the main channel. As soon as a single PT started to grow in the main channel, the pollen grain inlet was closed with a stopper and the inlet tubes to pump growth mediums were connected to the LoC device.

### Fluorescent Staining

Fluo‐3, Pentapotassium salt (ThermoFisher F3715) dye was used to stain extracellular Ca^2+^; while utilizing Ca Green (Calcium Green‐1, AM, cell permeant, C3011MP) dye to stain intracellular Ca^2+^. All the fluorescent experiments were conducted using Fluo‐3 to visualize the side streams in relation to the middle stream (i.e., interface detection), and CaGreen for PT detection, both under fluorescent microscopy.

### LoC Experiments

During the LoC experiments, inlet tubings of the LoC device were connected to glass syringes, containing the growth medium solutions having optimum Ca^2+^ (to form the middle stream) and rich (or poor) Ca^2+^ (to form side streams) and the flow rates were controlled utilizing a syringe pump (CETONI GmbH). For observation and recording, the LoC device was placed on an inverted fluorescent microscope (IX 71; Olympus) connected to a camera (Hamamatsu DUAL CCD CAMERA C11254). We waited until the pollen tube was in the main channel, and then started the continuous flow LoC experiments, performed either manually (at a constant TFR of 6–10 µL min^−1^) or with the automated (at constant TFR of 6–14 µL min^−1^) control. Experiments were recorded with various frame rates, i.e., one frame every 3–5 s. For the fluorescence experiments we used a 470 nm pulsing excitation and 505–535 nm emission filter. After completion of the experiments, the tubing and syringes were carefully rinsed with deionized water, and the LoC device with experiment materials was properly disposed as biological waste.

### Control experiments in LoC devices

To verify that PT turning responses were specifically triggered by changes in Ca^2+^ concentration–rather than by relative flow rate changes or the use of fluorescent dyes–control experiments were conducted. All control experiments were performed at a constant total flow rate (TFR) as the main experiments, maintained between 6–14 µL min^−1^.

*(i) Control Group I*: To assess the turning response in the absence of a Ca^2+^ gradient and rule out any influence from the interface between co‐flowing streams, the germination medium was pumped with optimal Ca^2+^ concentration (130 µm) through all inlets (Figure S13a, Supporting Information) and five PTs were observed. None showed any turning event (see green data points in Figures 2c) upon encountering the interface or when relative flow rates were changed, while keeping the TFR constant.

*(ii) Control Group II*: To verify that the Fluo‐3 dye had no impact on PT growth or turning, the germination medium including 130 µm Ca^2+^ and 1 µm Fluo‐3 was introduced through the side inlets, while the central inlets carried dye‐free germination medium including 130 µm Ca^2+^ (Figure S13b, Supporting Information) and five PTs were observed. None showed any turning event (see green data points in Figure 2h) upon encountering the interface or when relative flow rates were changed, while keeping the TFR constant.

*(iii) Control Group III*: To confirm that CaGreen also had no effect on PT growth or turning, the germination medium including 130 µm Ca^2+^ and 1 µm CaGreen was introduced through the side inlets, while the central inlets carried dye‐free germination medium including 130 µm Ca^2+^ (Figure S13c, Supporting Information) and four PTs were observed. None showed any turning event (see green data points in Figure 2c) upon encountering the interface or when relative flow rates were changed, while keeping the TFR constant.

### Characterization of Ca^2+^ Diffusion in LoC Devices

To measure the diffusion coefficient of Ca^2+^, first a standard (calibration) curve was obtained for Fluo‐3 (1 µm) in germination medium at pH 5.5 using a 96‐well spectrometer (see Figure S14, Supporting Information). The highest concentration of 10 mm Ca^2+^ was used and diluted with a 1:1 factor with milli‐Q water, until a Ca^2+^ concentration of 4.77×10^−6^ mm. This calibration allowed to evaluate the response of the Fluo‐3 to the Ca^2+^ concentrations in germination medium, since its response could vary significantly in solutions at different conditions, e.g., pH. Specifically, the range of Ca^2+^ concentrations (2×10^−6^ to 1×10^−3^ m) that showed a linear relationship between the fluorescence intensity and logarithm of concentrations, without any saturation, was identified. Accordingly, to perform the diffusion characterizations, the three different Ca^2+^ concentrations (2, 50, and 1000 µm) within the calibrated range were chosen, and three different growth medium solutions including various Ca^2+^ concentrations (2, 50, and 1000 µm) and 1 µm of Fluo‐3 dye were prepared. The LoC device (without loading any pollen grains) was connected to the syringe pump to form three co‐flowing streams of three different Ca^2+^ concentrations, such as 2 µm in the middle flow, and 50 and 1000 µm in two side flows (See also Figure S5, Supporting Information), forming a FRR of 0.33 (AFRR of 0.5) at a TFR of 450 µL min^−1^, and the fluorescent image of main channel was recorded. The fluorescent image of main channel was also recorded under the continuous flow (at a TFR of 450 µL min^−1^) of single growth medium solution having the 1000 µm Ca^2+^ as a control baseline to get rid of the background signal induced by illumination differences. The signal values at around 0.9 mm from the junction point of the streams were measured along the width of the channel for both images including experimental data and the control baseline (Figure S5a,b, Supporting Information). The actual signals were calculated by subtracting the baseline signals from the obtained apparent signals (Figure S5c,d, Supporting Information). The actual signals were averaged at the center position of each stream to obtain the exact corresponding signal values for the known Ca^2+^ concentrations (2, 50, and 1000 µm), avoiding the diffusion regions at the interfaces of different streams. A linear fit (using Origin software) was performed between the logarithm of these known concentrations and their corresponding signal values to form a calibration curve (Figure S5e, Supporting Information) and convert other signal values to their actual concentrations, resulting in a concentration profile along the diffusion direction (Figure S5f, Supporting Information). This data was then compared to the results of numerical simulations to determine the diffusion coefficient of the Ca^2+^ in the growth medium solution (see also details on *Numerical Simulations*).

### Rheological Characterizations

Viscosity measurements of the growth medium were conducted using an Anton Paar MCR 501 rheometer, equipped with a PP25‐rough geometry. The dependence of viscosity on shear rate was analyzed by subjecting the medium to a range of shear rates (from 1 to 1000 s^−1^) at room temperature, maintaining a constant gap height of 0.5 mm. For density measurements, the growth medium was carefully introduced into a U‐shaped glass capillary, and the density was subsequently measured utilizing Anton Paar/DMA 500 density meter. These measurements were repeated three times to obtaining the average value and standard deviations.

### Numerical Simulations

To better understand the Ca^2+^ gradients prevailing around the PT tip, numerical simulations of the fluid flow and mass transport within the LoC device were performed. The rheological parameters of the relevant fluids (i.e., density and viscosity of growth mediums with and without calcium) were obtained experimentally and then used in the numerical simulations (Figure S3, Supporting Information). In preliminary hydrodynamic simulations performed for a TFR of 6 µL min^−1^, it was found that the shear rates within the LoC device range between 30 (at center) and 900 s^−1^ (near walls). Because the performed rheological characterizations showed that the growth media behave as Newtonian fluids for the mentioned shear rates (Figure S3b, Supporting Information), it was assumed in the simulation that the growth media had constant viscosity. The flow rates and Ca^2+^ concentrations at the inlet were set based on the conditions used in typical LoC experiments, whereas atmospheric pressure was assumed to prevail at the outlets. The velocity of the fluid at the walls of the device and at the PT was assumed to be null (i.e.*, “no‐slip”* condition). Velocity, pressure and Ca^2+^ concentration were calculated using the finite volume method by coupling the Navier‐Stokes equation, the continuity equation, and the species transport equation that are respectively given by:

(11) $$\frac{\partial \vec{V}}{\partial t}+\vec{V}\left(\nabla \cdot \vec{V}\right)=-\frac{1}{\rho }\nabla P+\upsilon \nabla^{2}\vec{V}$$

(12) $$\frac{\partial \rho }{\partial t}+\nabla \left(\rho \vec{V}\right)=0$$

(13) $$\frac{\partial \left(\rho Y_{i}\right)}{\partial t}+\nabla \left(\rho \vec{V}Y_{i}\right)=\rho D_{i}\;\nabla^{2}Y_{i}$$

where overrightarrowV is the velocity vector, ∇ is the divergence operator, ρ is the density, P is the pressure, υ is the viscosity, ∇^2^ is the Laplacian operator, *Y_i_* is the mass fraction of species i and *D_i_* is the diffusion coefficient of species i. The simulations were performed in steady‐state, employing the SIMPLE algorithm for velocity‐pressure coupling and second order upwind discretization. The simulations were assumed to be converged when residuals were less than 10^−5^, given that simulations performed with stricter criteria produced similar results (Figure S15, Supporting Information). Furthermore, simulations to determine the coarsest mesh that produced mesh‐independent results were conducted. It was found that a mesh containing around 4.6 million cells (half of its cross‐section is shown in Figure S4a,b, Supporting Information) produced calcium concentration profiles similar to those obtained using a much finer mesh (around 10.5 million, see Figure S4c, Supporting Information). Therefore, the former mesh was found to be adequate for simulating the transport of calcium in the LoC device. The flow and mass transport predictions obtained via numerical simulation were validated by comparison with the respective analytical solutions.^[^ ^63^, ^64^ ^]^ As shown in Figure S4d,e (Supporting Information), good agreement between the numerical predictions and the analytical solutions was found, which demonstrated that the numerical approach followed in the present work can be used to accurately predict the fluid flow and mass transport occurring in the LoC device. Lastly, the diffusion coefficient of Ca^2+^ in the growth medium was studied experimentally by measuring fluorescence intensity (see *Characterization of Ca^2+^ Diffusion in LoC Devices*). These experiments were replicated in numerical simulations considering two values for the diffusion coefficient of calcium: 1.3×10^−9^ (from literature^[^ ^65^ ^]^) and 7×10^−9^ m^2^ s^−1^ (maximum value that fitted the experimentally obtained fluorescence intensity curves). As it was found that the two diffusion coefficients tested produce rather similar calcium concentration profiles along the width of the device, it was chosen to simply use the value that showed a good agreement with the experimental data (Figure S5f, Supporting Information) and was referenced in the literature (1.3×10^−9^ m^2^ s^−1^) in all subsequent simulations.

### Brightfield Pollen Tube Tracking

The microscopy images in brightfield are poor in texture and low in signal‐to‐noise ratio, leading to insufficient performance of classical feature‐based object tracking methods because of the degradation of traditional feature descriptors.^[^ ^66^, ^67^ ^]^ Moreover, the pollen tube is a growing living cell, which is even more challenging to detect in real time compared to normal translating or rotating objects. Therefore, a fusion of various tracking methods, such as contour detecting, background subtraction motion tracking and optical flow, were utilized for a robust performance^[^ ^68^, ^69^ ^]^(Figure S10, Supporting Information). More precisely, the real‐time frame is first pre‐processed for noise elimination, and then optical flow is applied to the frame to find out magnitude and orientation of movement within the image. In microscopic scenes, most of the objects are still (background) while the PTs are moving (i.e., growing). Therefore, when the optical flow magnitude is small, we consider the PT growth is small, and then go on to contour tracking to localize the tip position and orientation by following steps of Canny edge detection, convex contour recognition, and ellipse fitting, respectively. If, on the other hand, optical flow analysis outputs relatively large magnitude, we apply a KNN‐based background subtractor to the image to specify the moving elements, and then use DBSCAN to exclude noises and precisely localize the tip of PT. In this way, we could take advantages of both tracking methods and accomplish robust PT tracking in real time under brightfield microscopy.

### Automated Setup Development

The automated setup was developed in LabVIEW 2019. CETONI SDK Software Development Kit for LabVIEW integration was used to connect and control the pump. LabVIEW Python functions were used to integrate Python codes in LabVIEW. PCC was built on Python 3.6 with various public libraries including OpenCV 3.4.2 and scikit‐learn 0.24.2. The software code is available in the public GitHub repository (https://github.com/HeyyFrank/Automated‐LoC‐Approach‐for‐Pollen‐Tube‐Growth‐Manipulation.git).

### Statistical Analysis

Statistical data were presented in boxplot. The box, whiskers, square, line and dots represent the range of 25–75% of the data, 1.5 inter quartile range, mean, median and outliers of the data. Sample size of each statistical analysis was listed in the figure legend. OriginPro 2024 was used for statistical analysis. Moreover, the error bars in the other graphs represent ± standard deviation, with the sample size (n) specified in the corresponding figure legend.

## Conflict of Interest

The authors declare no conflict of interest.

## Author Contributions

S.S., B.J.N., and U.G. conceived the concept, designed, and supervised the study; H.V. and S.P. contributed to methodology development and supervision of the study; J.Z. designed and fabricated the LoCs, developed the automated setup and experimentation pipeline, analyzed and visualized the results; M.B. conducted the manual and automated LoC experiments, and processed the data; J.P.V. and T.S.M. conducted numerical simulations; V.P. and P.F. conducted the rheological characterizations; J.Z., M.B., S.P., and S.S. wrote the original draft. All authors contributed to discussion and writing the final version of manuscript.

## Supporting information



Supporting Information

Supplemental Movie 1

Supplemental Movie 2

Supplemental Movie 3

Supplemental Movie 4

Supplemental Movie 5

Supplemental Movie 6

Supplemental Movie 7

Supplemental Movie 8

Supplemental Movie 9

Supplemental Movie 10

Supplemental Movie 11

## Data Availability

All data needed to evaluate the conclusions in this work are available in the main text and in the Supplementary Information. Additional data related to this paper may be requested from corresponding authors. The software code is available in the public GitHub repository (https://github.com/HeyyFrank/Automated‐LoC‐Approach‐for‐Pollen‐Tube‐Growth‐Manipulation.git).
