# Supplementary material for: An Automated Lab‐On‐A‐Chip Approach for Pollen Tube Growth Manipulation in a Controlled Chemical Environment
Source: Adv Sci (Weinh). 2025 Sep 29;12(43):e07434. doi: 10.1002/advs.202507434 (PMC12631934; doi:10.1002/advs.202507434)
Supplement: Supplementary file 1 — Supporting Information [file ADVS-12-e07434-s006.pdf]

Supporting Information

An automated lab-on-a-chip approach for pollen tube growth manipulation in a controlled chemical environment

Jiawei Zhu, Marta Belloli, João P. Vale, Vitaly Pustovalov, Peter Fischer, Hannes Vogler, Tiago S. Mayor, Salvador Pané, Semih Sevim, Ueli Grossniklaus*, Bradley J. Nelson**

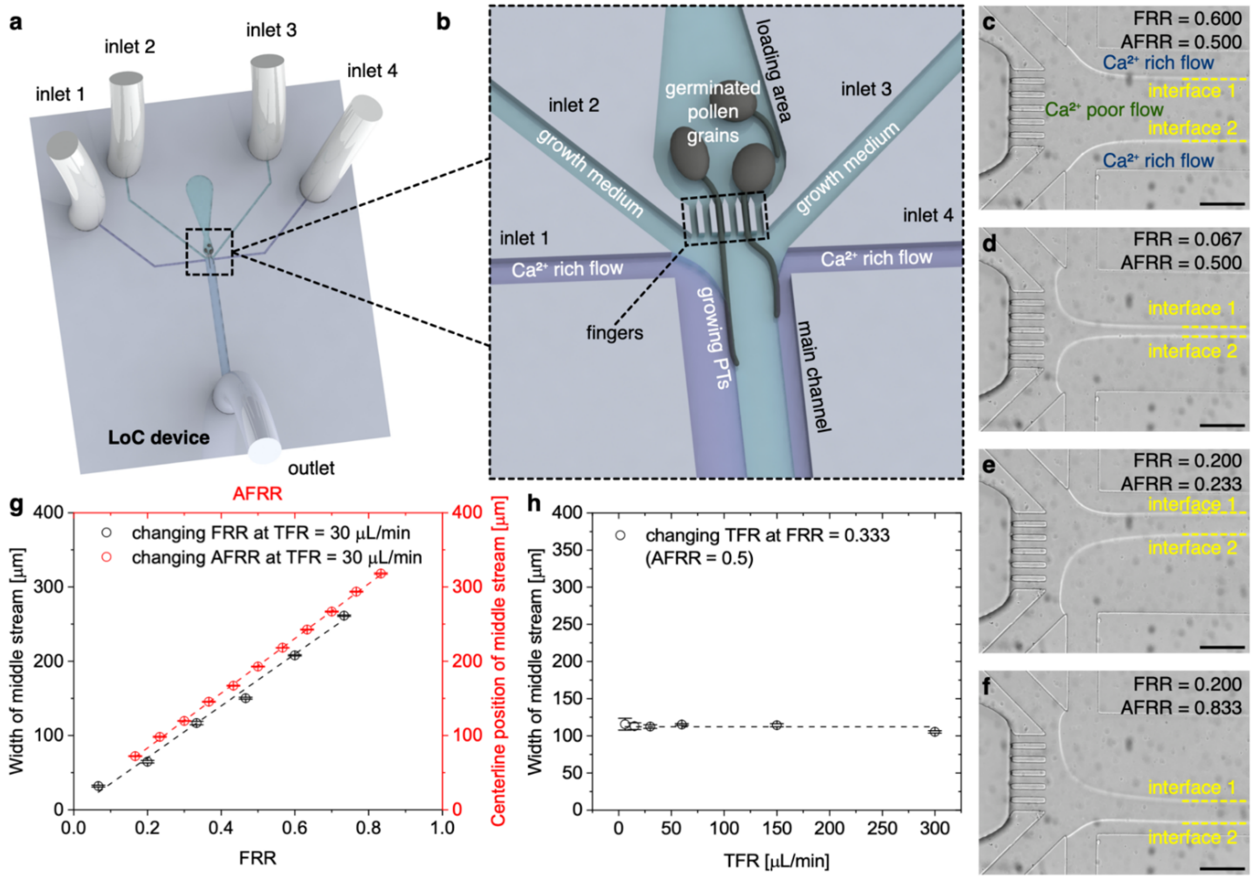

Figure S1. Design and hydrodynamic characterization of LoC devices. (a) Schematic design of our LoC device. (b) Illustration showing the detailed design of the LoC device and schematic representation of the approach to control the Ca^{2+} concentrations around the tip of a growing PT. (c-f) Hydrodynamic flow focusing experiments showing the various interface positions at different FRRs and AFRRs, while keeping TFR constant at 30 µL/min. Scale bars are 150 µm. (see also **Supporting Movie 1**) (g) Dependency of the width and centerline position of the middle stream to the FRR (in black) and AFRR (in red), respectively, within our LoC device having a 400 µm-wide main channel. Note that while changing the FRR (in black), the AFRR were kept constant at 0.5; and while changing the AFRR (in red), the FRR were kept constant at 0.2. Both sets were performed at a constant TFR of 30 µL/min. (h) Dependency of the width of middle stream to TFR while keeping both FRR and AFRR constat at 0.333 and 0.5, respectively. The error bars in panel (g) and (h) represent ±standard deviation for five measurements along the channel length.

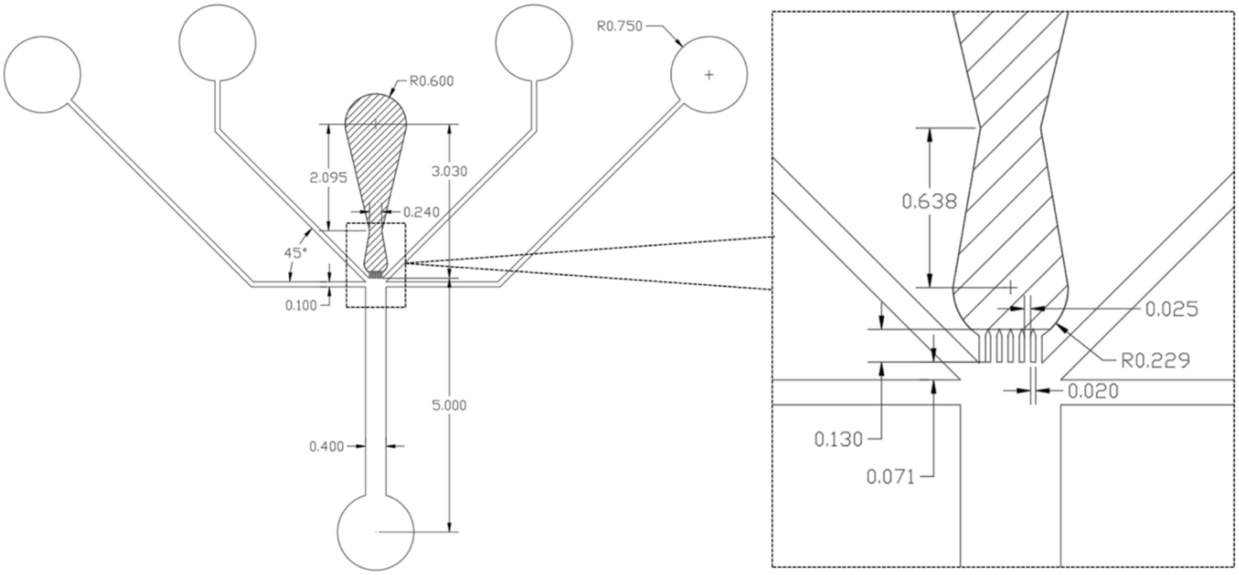

Figure S2. CAD design of the LoC devices. Technical drawing showing the design of microfluidic device utilized in this work. Dimensions are in millimeter. Hatched area has a height of 130 µm to load pollen grains, whereas the rest of the channels has a height of 35 µM.

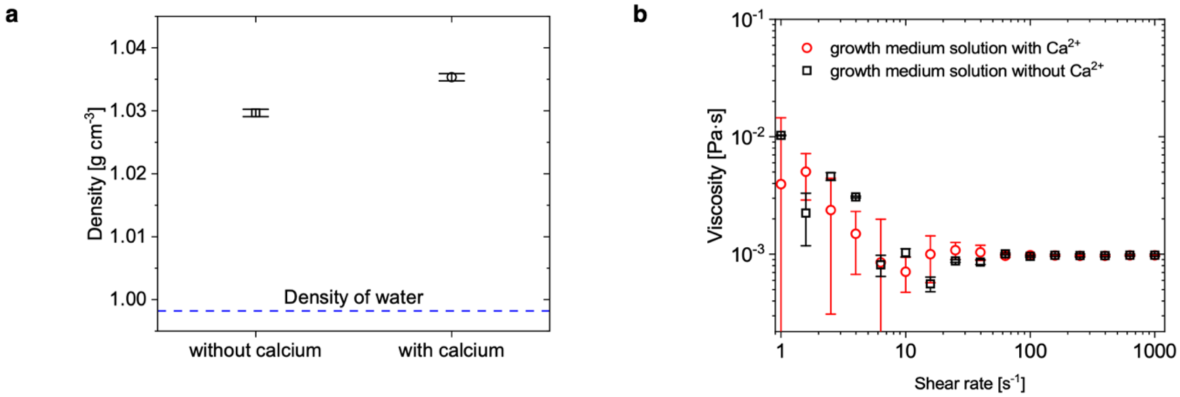

Figure S3. Rheological characterization of growth mediums. (a) Density measurements of growth mediums with and without Ca^{2+} (b) Viscosity of growth mediums with (red) and without (black) Ca^{2+} based on the applied shear rates (from 1 to 1000 s^{-1}). The error bars represent the ±standard deviation.

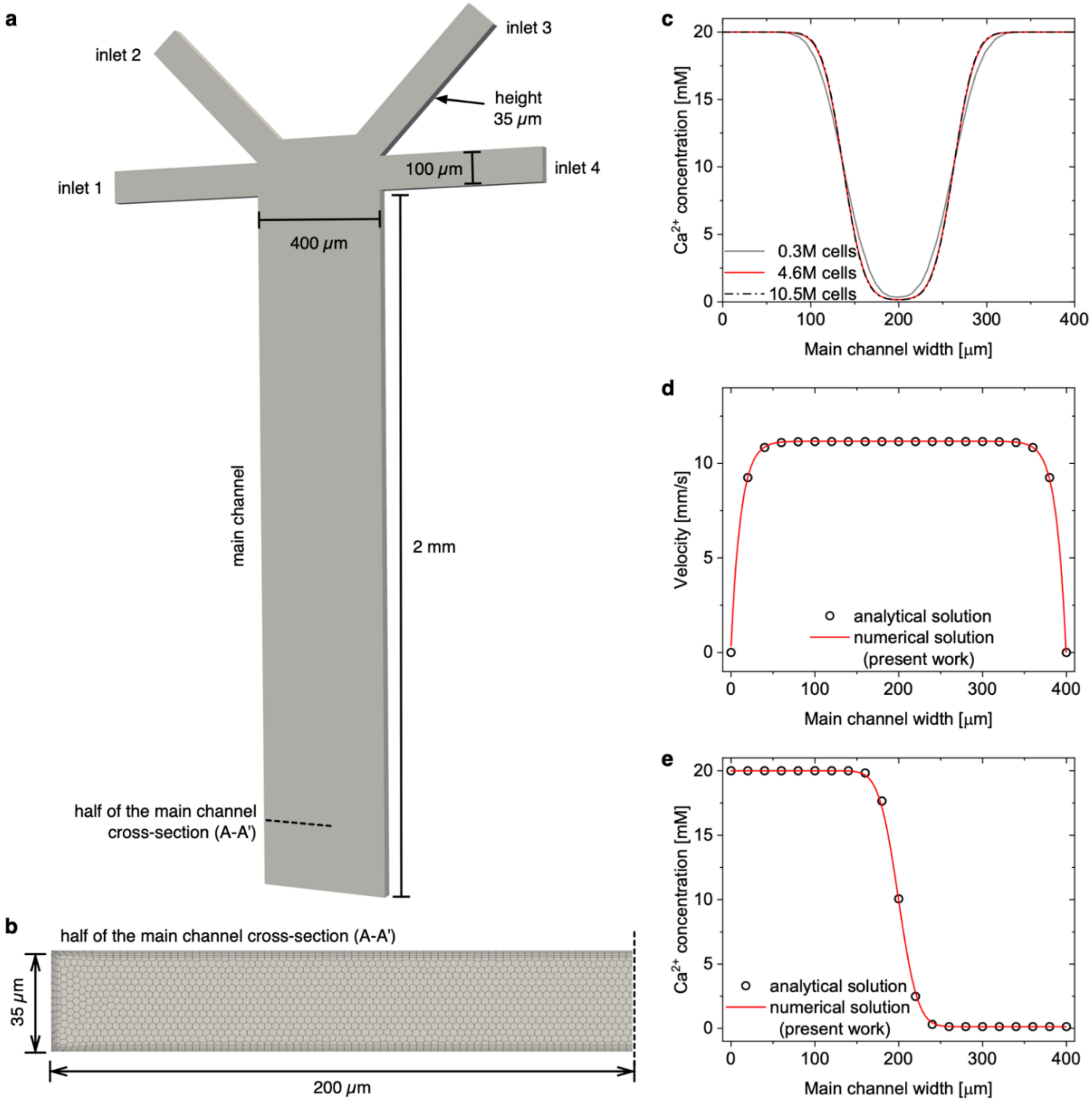

Figure S4. Validation of numerical simulations. (a) Simulation domain. (b) Representative mesh used in simulations shown on half of the cross-section of main channel. (c) Ca^{2+} concentration profiles along the main channel width obtained from numerical simulations performed for TFR = 6 μL/min with different meshes (0.3, 4.6, and 10.5 million cells, in grey solid, red solid, and black dash-dotted lines, respectively). (d-e) Comparison of analytical solution (black circles) and numerical solution (red line) for the (d) velocity and (e) Ca^{2+} concentration profiles along the main channel width for TFR = 6 μL/min.

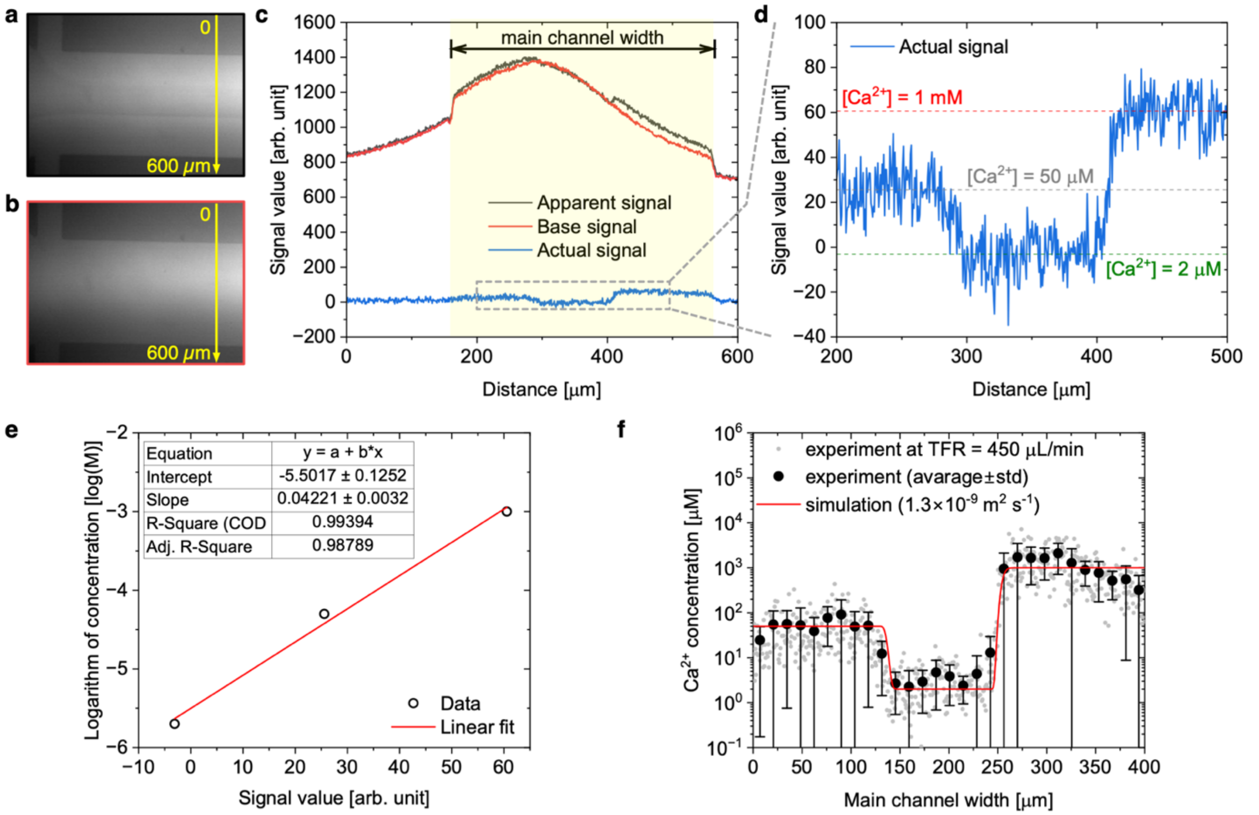

Figure S5. Characterization of Ca^{2+} diffusion in LoC devices. (a) Fluorescent image of main channel of LoC device showing the formation of three co-flowing streams with three different Ca^{2+} concentrations, such as 2 μM in the middle flow, and 50 μM and 1000 μM in two side flows, forming a FRR of 0.33 (AFRR of 0.5) at a TFR of 450 μL/min. (b) Fluorescent image of main channel of LoC device under the continuous flow (at a TFR of 450 μL/min) of single growth medium solution having the 1000 μM Ca^{2+} as a control baseline to get rid of the background signal induced by illumination differences. (c) Graph showing the apparent (black), base (red) and actual (blue) signals and (d) magnified actual signal in the main channel area. (e) A linear fit between the logarithm of concentrations and their corresponding signal values to form a calibration curve. (f) Comparison of the numerical simulation (red line corresponding to a simulation assuming the diffusion coefficient of calcium as 1.3×10^{-9} m^2/s) and experimental results (grey dots are the individual measurements, black dots are an average ± standard deviation) showing the Ca^{2+} concentration profile along the main channel.

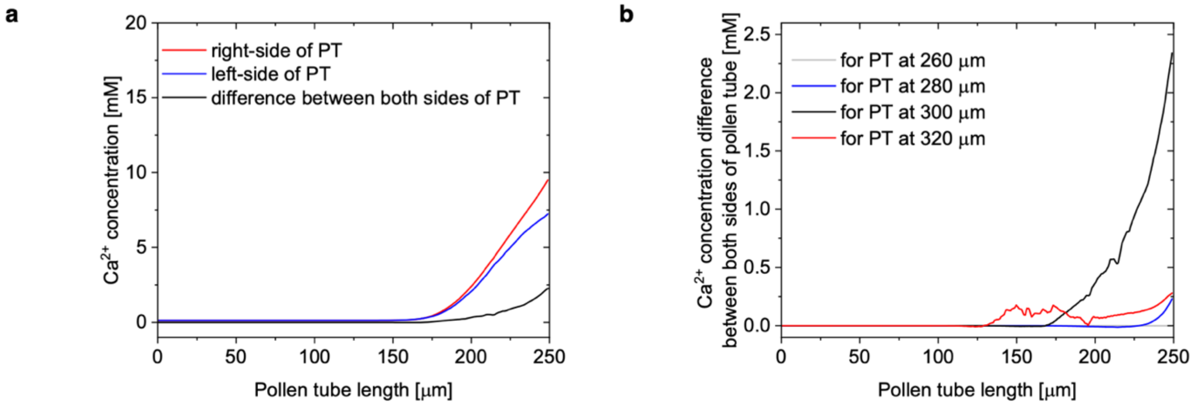

Figure S6. Ca^{2+} concentration difference along the right- and left-side of the pollen tube length. (a) For a PT tip placed at the interface between the optimal (130 μM) and calcium rich (20 mM) growth mediums, attaining a FRR of 0.33 (AFRR of 0.5) at TFR of 10 μL/min. (b) Same scenario at different PT tip positions with respect to the interface (see also **Figure 3e**).

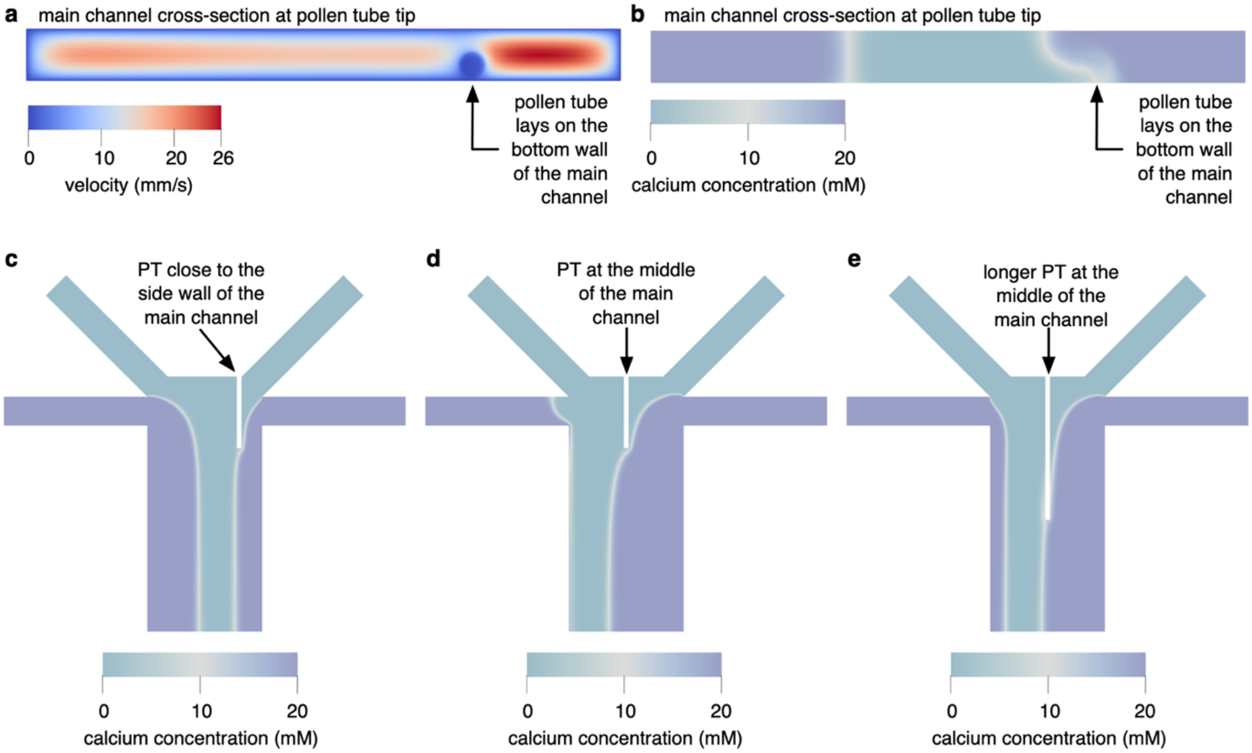

Figure S7. Numerical simulations considering various positions of PT within the main channel. (a) Velocity and (b) concentration maps of the main channel's cross-section showing the results around the PT tip, which lays on the bottom wall of the LoC device. (c-e) Calcium concentration maps for PT of various lengths growing in different positions along the width of the device for a FRR of 0.33 at a TFR of 10 µL/min. (c) PT at 320 µm and AFRR is 0.389, (d) PT at 200 µm and AFRR is 0.773, as well as (e) PT with twice the length (500 µM) at 200 µm, and AFRR is 0.700.

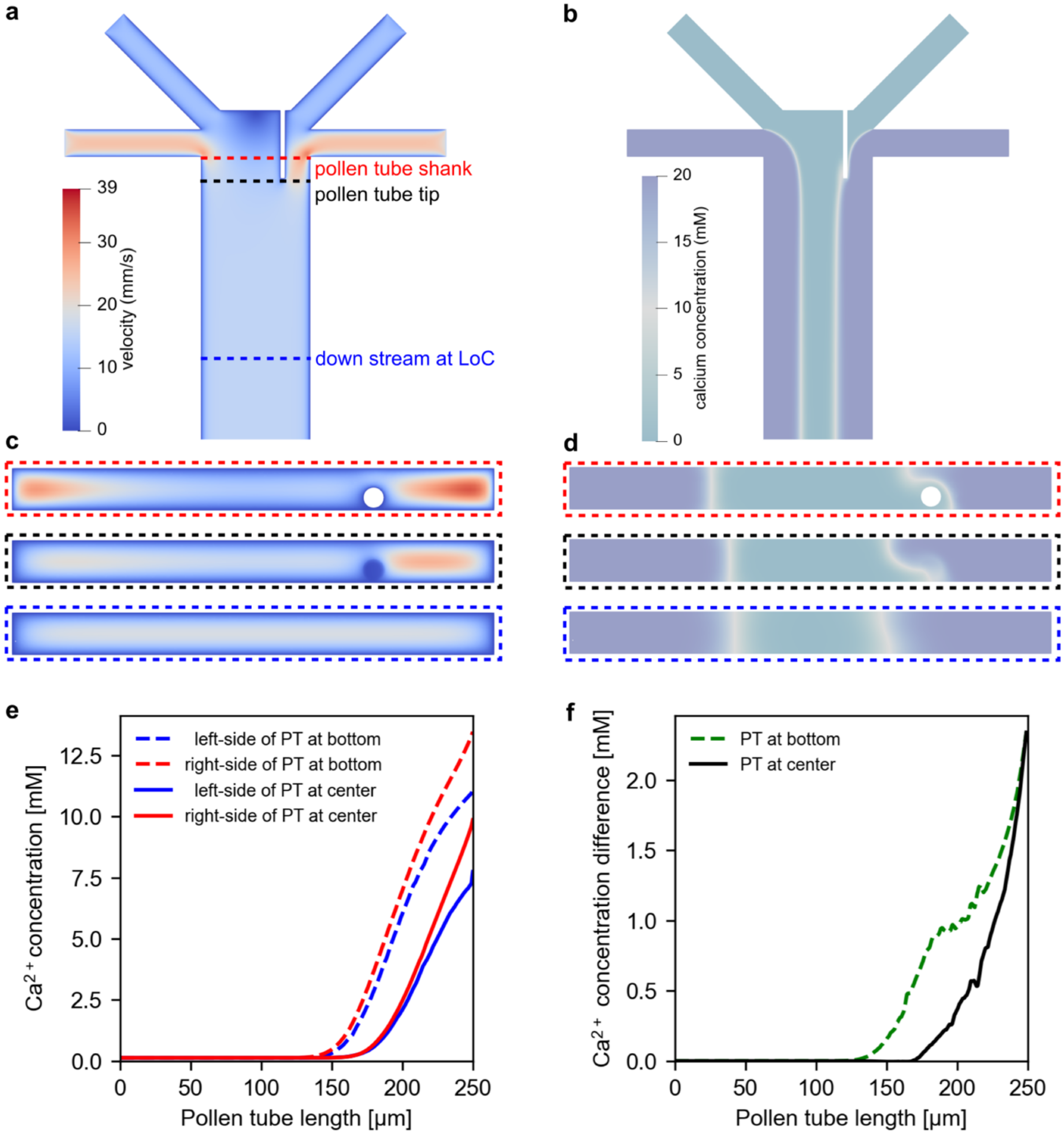

Figure S8. Numerical simulation considering the PT to be at 2 μm away from the bottom wall. (a-b) Velocity and concentration maps at the middle of the height of the PT. (c-d) Velocity and concentration maps at different positions along the length of the device. (e) Ca^{2+} concentration along right- and left-side of PT when it is placed near the bottom wall or at the center. (f) Ca^{2+} concentration different (right-left) along PT length when it is placed near the bottom wall or at the center.

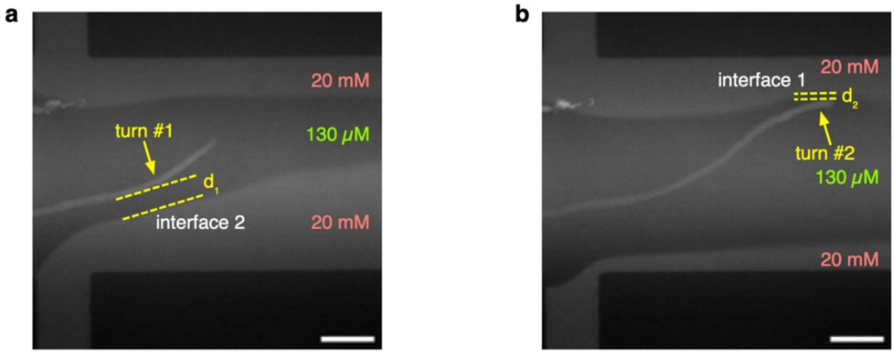

Figure S9. Turning events at different interface-to-PT-tip distances. (a-b) Fluorescence microscopy images showing different turning events in manual LoC experiments performed with the calcium rich growth medium (20 mM of Ca^{2+} concentration), while the interface-to-PT-tip distance is (a) large and (b) small, respectively. Scale bars are 100 µm. See also **Supporting Movie 7**.

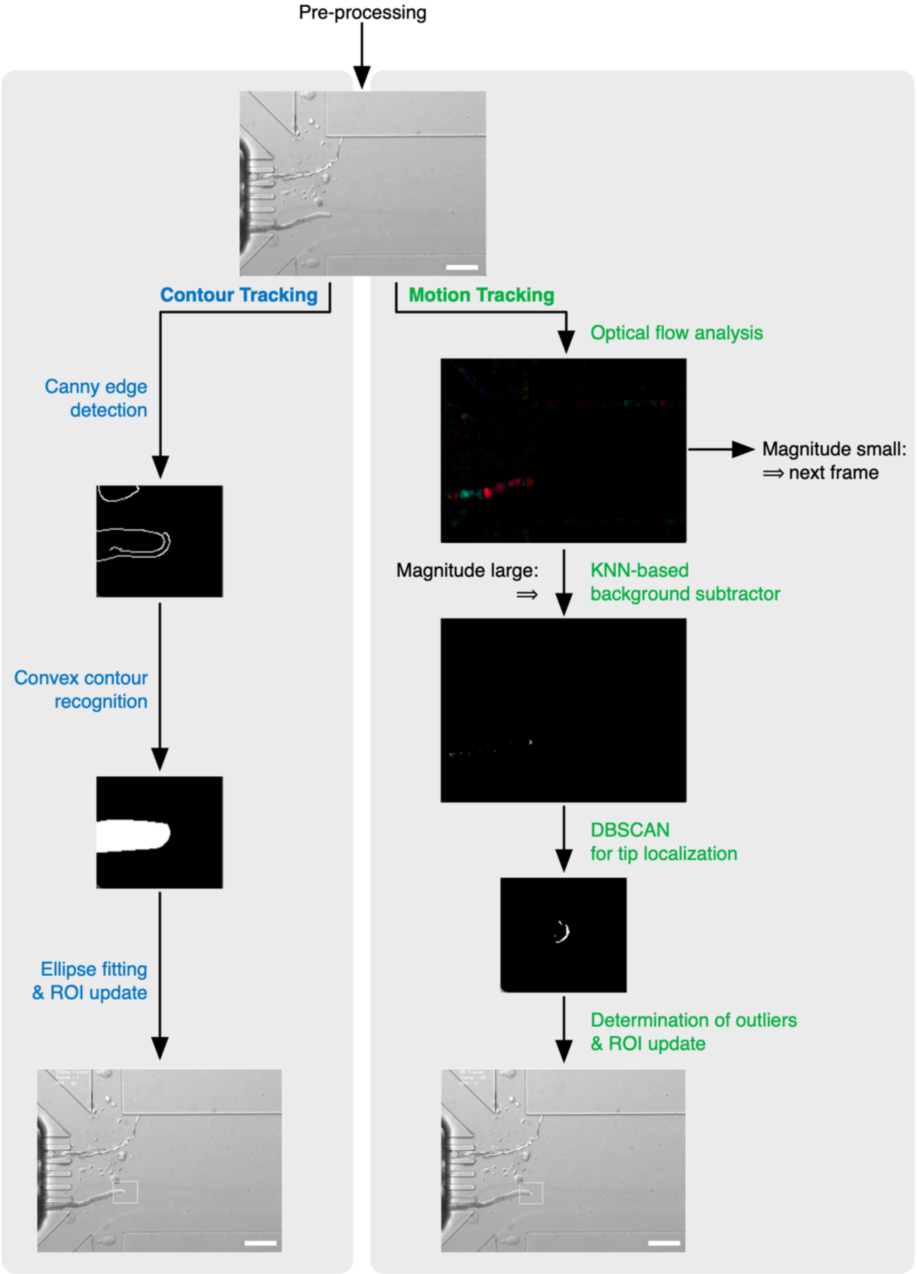

Figure S10. Workflow of brightfield PT tracking. Showing different methodologies for data analysis of manual LoC experiments. Scale bars are 100 µm.

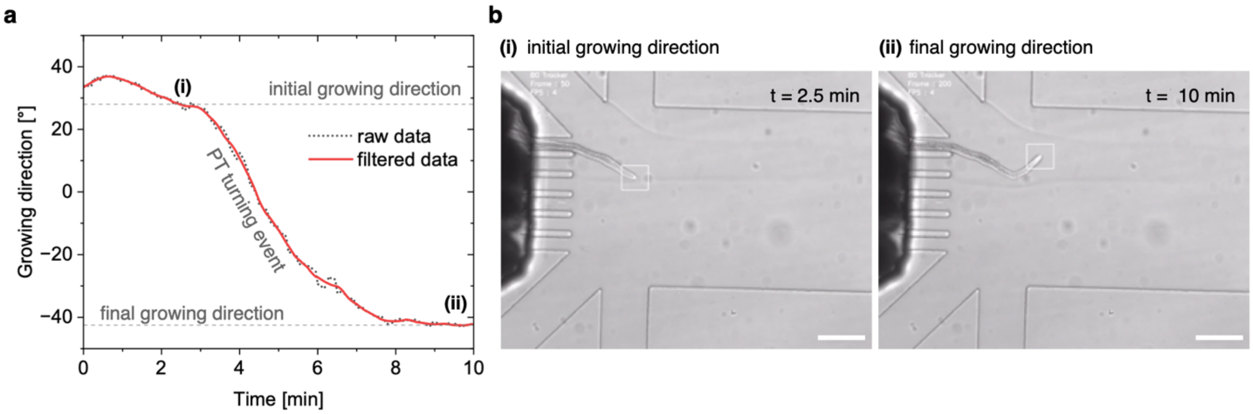

Figure S11. Brightfield PT tracking in manual LoC experiments. (a) Collected and processed growing direction data by the tracker for analysis of turning angle. (b) Brightfield images showing the PT tracking during the manual LoC experiment at (i) t = 2.5 min and (ii) t = 10 min, where the growing PT tip subjected to the interface between optimum (130 µM) and calcium rich (7.5 mM) growth medium. Scale bars are 100 µm. See also **Supporting Movie 8**.

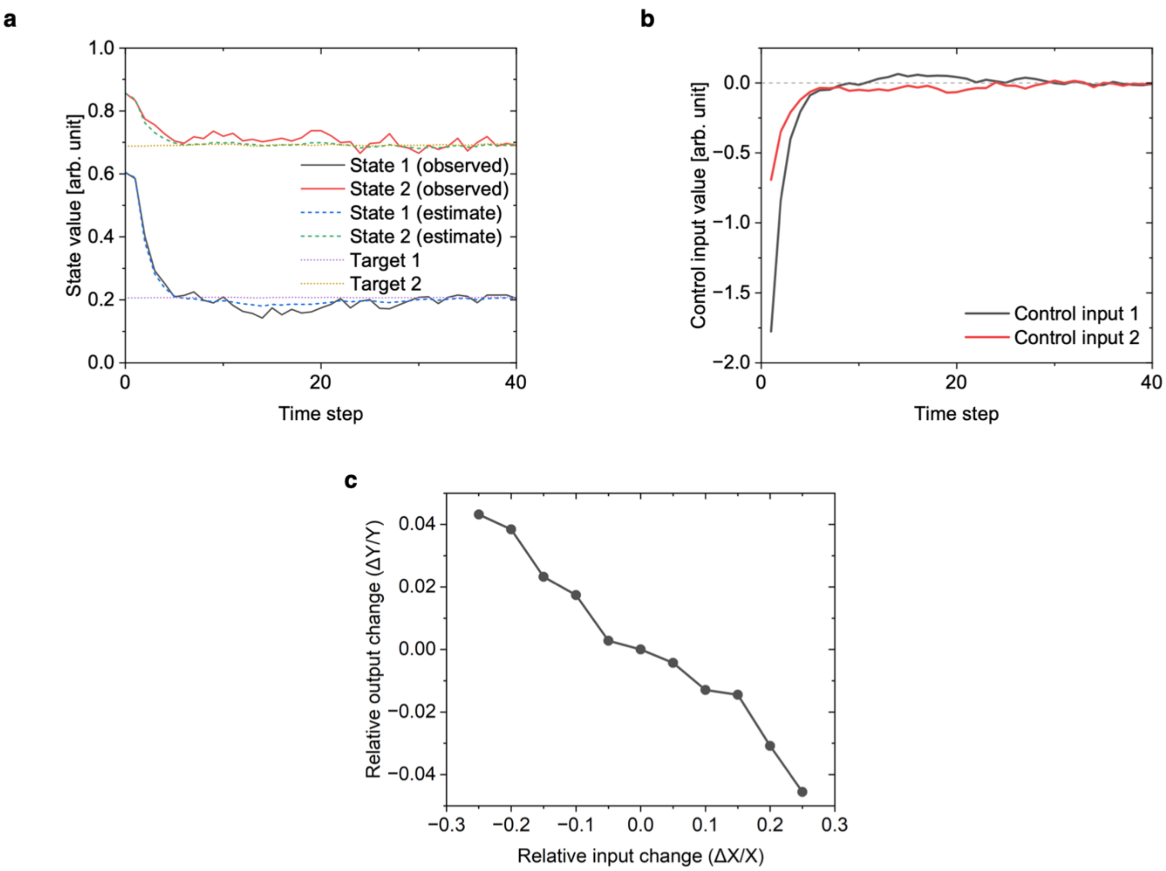

Figure S12. Computational test simulations to evaluate the controller performance and sensitivity analysis. (a) Simulated state evolution using our developed controller, including observed states with added random noise, estimated states by the Kalman filter, and target states. (b) Simulated control inputs using the developed controller. (c) Sensitivity analysis of the developed controller, demonstrating a linear relationship between the relative output change and relative input change with a ratio of approximately 0.16, when varying the control inputs from −25% to +25% relative to their calculated values by the controller.

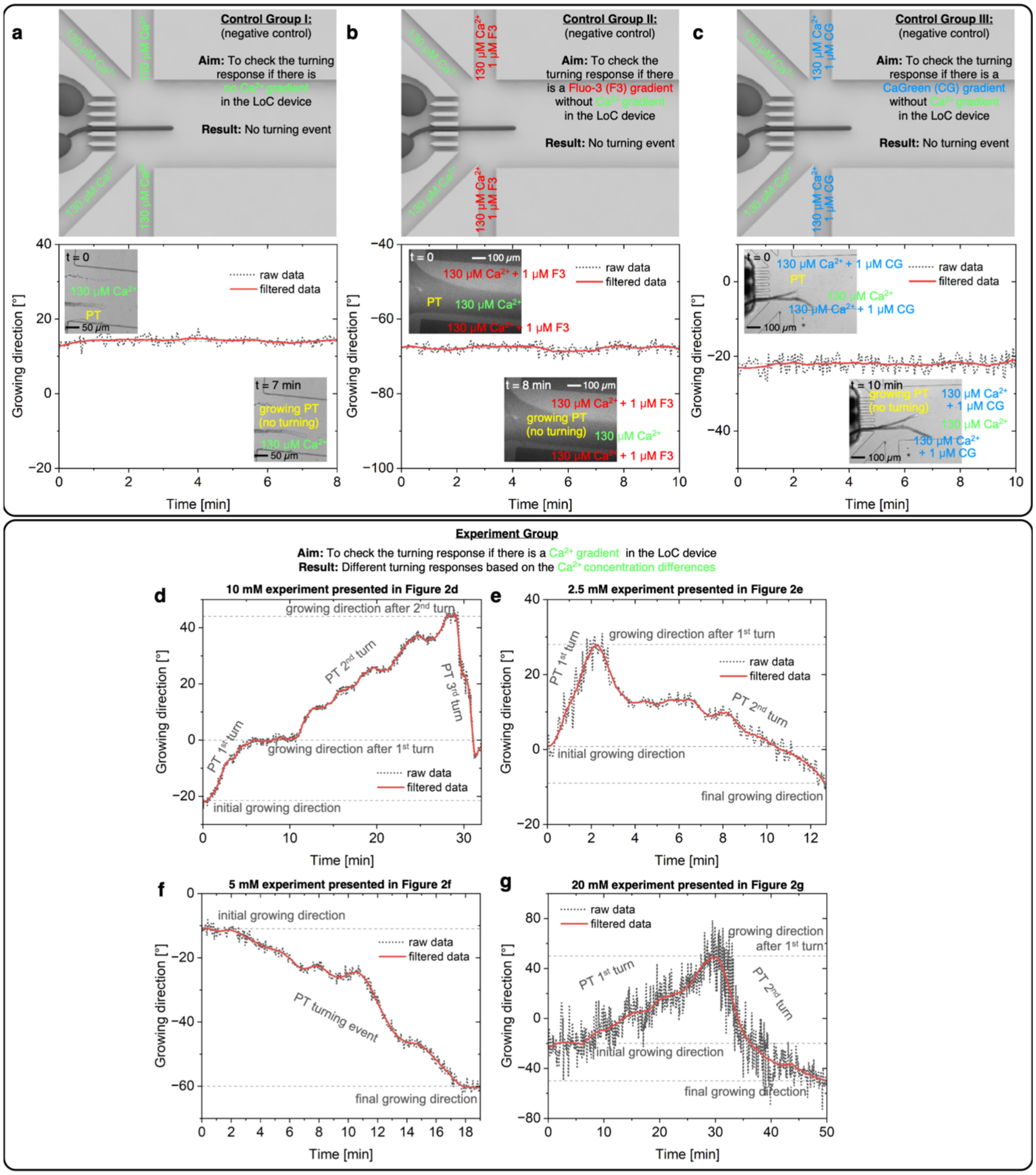

Figure S13. Details of control experiments performed in LoC devices and quantitative analysis of PT growth in experimental groups. (a) Control Group I: Schematic representation of control experiments in which germination medium containing 130 μM Ca^{2+} was pumped from all inlets to test the turning response in the absence of a calcium gradient. The representative analysis (graph and corresponding bright-field images in bottom panel) shows that no turning events occurred when no Ca^{2+} concentration difference was present. (b) Control Group II: Schematic representation of control experiments where the side streams contained germination medium with 130 μM Ca^{2+} and 1 μM Fluo-3, while the central stream carried dye-free germination medium with 130 μM Ca^{2+}. The representative analysis (graph and corresponding fluorescence microscopy images in bottom panel) shows that no turning events occurred in response to Fluo-3 dye gradients in the absence of a Ca^{2+} gradient. (c) Control Group III: Schematic representation of control experiments where the side streams contained germination medium with 130 μM Ca^{2+} and 1 μM CaGreen, while the central stream carried dye-free germination medium with 130 μM Ca^{2+}. The representative analysis (graph and corresponding bright-field images in bottom panel) confirms that CaGreen dye gradients, without a Ca^{2+} gradient, did not trigger any turning events. (d–g) Analyzed and quantified PT growth and turning responses presented in **Supporting Movies 3, 4, 5** and **6**, respectively.

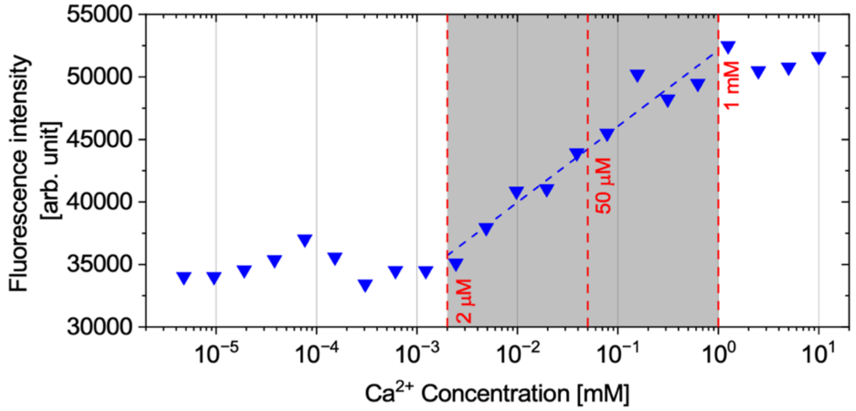

Figure S14. Calibration curve of Fluo-3 in growth medium at pH 5.5. Blue dashed lines represent the linear trend of the calibration curve between 2 µM and 1 mM, whereas the vertical red dashed lines mark the calcium concentrations (2 µM, 50 µM, and 1mM) used in the microfluidic experiment presented in **Figure S5**.

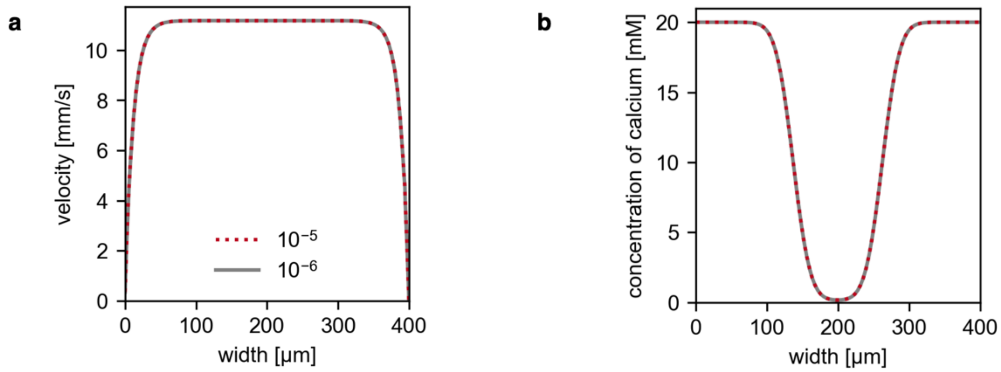

Figure S15. Numerical simulations for convergence criteria analysis. (a)Velocity and (b) concentration profiles are displayed on the left and rightmost plots, respectively. These simulations were performed for TFR = 6 μL/min and a Ca^{2+} concentration of 20 mM from inlets 1 and 4, which is similar to the mesh testing analysis previously performed and shown in **Figure S4**.
